# Transcription factor-based biosensors enlightened by the analyte

**DOI:** 10.3389/fmicb.2015.00648

**Published:** 2015-07-01

**Authors:** Raul Fernandez-López, Raul Ruiz, Fernando de la Cruz, Gabriel Moncalián

**Affiliations:** Departamento de Biología Molecular and Instituto de Biomedicina y Biotecnología de Cantabria, Universidad de Cantabria – Consejo Superior de Investigaciones CientíficasSantander, Spain

**Keywords:** biosensors, transcription factor, effector, aromatic compounds, metal, analyte

## Abstract

Whole cell biosensors (WCBs) have multiple applications for environmental monitoring, detecting a wide range of pollutants. WCBs depend critically on the sensitivity and specificity of the transcription factor (TF) used to detect the analyte. We describe the mechanism of regulation and the structural and biochemical properties of TF families that are used, or could be used, for the development of environmental WCBs. Focusing on the chemical nature of the analyte, we review TFs that respond to aromatic compounds (XylS-AraC, XylR-NtrC, and LysR), metal ions (MerR, ArsR, DtxR, Fur, and NikR) or antibiotics (TetR and MarR). Analyzing the structural domains involved in DNA recognition, we highlight the similitudes in the DNA binding domains (DBDs) of these TF families. Opposite to DBDs, the wide range of analytes detected by TFs results in a diversity of structures at the effector binding domain. The modular architecture of TFs opens the possibility of engineering TFs with hybrid DNA and effector specificities. Yet, the lack of a crisp correlation between structural domains and specific functions makes this a challenging task.

## General Design of Transcription Factor-Based Whole Cell Biosensors (WCBs)

Whole cell biosensors (WCBs) are devices that use specific biochemical reactions mediated by whole cells to detect chemical compounds usually by optical signals. WCBs are especially useful for environmental monitoring, as they are able to detect a wide range of pollutants in a very specific manner [for a review on methodologies to create WCBs and recent applications see Michelini et al. (2013) and Park et al. (2013)].

Bacteria contain transcription factors (TFs) able to respond to a wide variety of chemical signals. Thus, using genetic engineering, these TFs can be coupled to reporter genes (like fluorescent proteins or luciferases) to create WCBs. Although the use of TF-based biosensors was proposed years ago, few reliable systems have been developed so far. A summary of available TF/analyte pairs can be found in (Landrain et al., 2009). The emergence of synthetic biology, which intends to create synthetic devices able to perform input-sensing and biocomputing functions (Macia and Sole, 2014), has renewed the interest in TF-based bionsensors. In principle, implementing TF-based biosensing in a synthetic circuit is a simple task. Select the chemical analyte for the circuit to respond to, identify the correct TF that responds to that particular analyte, and make the expression of the responding gene (either a reporter gene or the next element in the logical process of the circuit) dependent upon the given TF. Unfortunately, this over – simplistic scheme rarely works, for bacterial TFs employ different mechanisms of analyte recognition and promoter activation/repression, which complicate considerably the development of functional devices. In this review we summarize the mechanisms of analyte recognition and transcriptional control of the most common TF families employed in biosensor development. We have classified them depending on the chemical nature of the analyte detected, thus bringing the input signal to center stage.

Whole cell biosensors are composed of two protein modules that can be combined depending on the analyte to detect and the output signal to obtain. The sensing module is the signal transducer, responsible for recognition of the analyte and transduction of this signal to the reporter module. The reporter module produces a measurable output (typically light, fluorescence, or color changes), depending on the state of the sensing module. Three main reporter modules are used for the construction of WCBs: luminescent enzymes [encoded by eukaryotic *luc* genes or bacterial *lux* genes (Wiles et al., 2009)], fluorescent proteins [green fluorescent protein (*gfp*) and its fluorescent variants (Shimomura, 1979; Chalfie et al., 1994)] and β-galactosidase (*lacZ*, Fowler and Zabin, 1978; Kalnins et al., 1983). Reporter modules have been extensively analyzed (Ghim et al., 2010; French et al., 2011; Shin et al., 2011; Gutiérrez et al., 2015) and will not be discussed in this review. In general, TF-based signal transducers can be combined with any of the aforementioned reporter systems. A detailed comparison between their usefulness and suitability for different applications can be found in (Hakkila et al., 2002).

In contrast with the limited repertoire of reporter genes, the variety of signal transducers in nature is enormous. Prokaryotes transform environmental signals to cellular responses using one-component or two-component systems. In two-component system, a membrane-bound sensor histidine kinase catalyzes its autophosphorylation and then transfers the phosphoryl group to a response regulator, which regulates gene expression (Laub and Goulian, 2007). The homology of the histidine kinase domains allows swapping of these domains and their cognate regulators to create chimeric systems (Ninfa, 2010). Some whole-cell biosensors were designed by using two-component systems, detecting input signals such as light, oxygen, or osmolarity changes (Zhang and Keasling, 2011). However, because typical two-component systems use kinase phosphorylation for module communication, undesired crosstalk between systems could happen, especially after overexpression of either a chimeric input domain or an unnatural response regulator. Nevertheless, the majority of signal transduction systems in bacteria consist of a single protein that contains both the input and output domains. These one-component systems display a greater domain diversity than two-component systems (Ulrich et al., 2005). One-component TF typically contain a DNA-binding domain (DBD), responsible for recognition and binding of the operator DNA, and an effector-binding domain (EBD), responsible for oligomerization of the regulator and transmission of the effector signal to the DNA-binding domain. Thus, one-component TFs are more versatile than two-component systems although, in the former, it is more complicated to swap EBDs and DBDs to create chimeric systems. Hereafter, TF will be used to refer to one-component TF.

**FIGURE 1:**
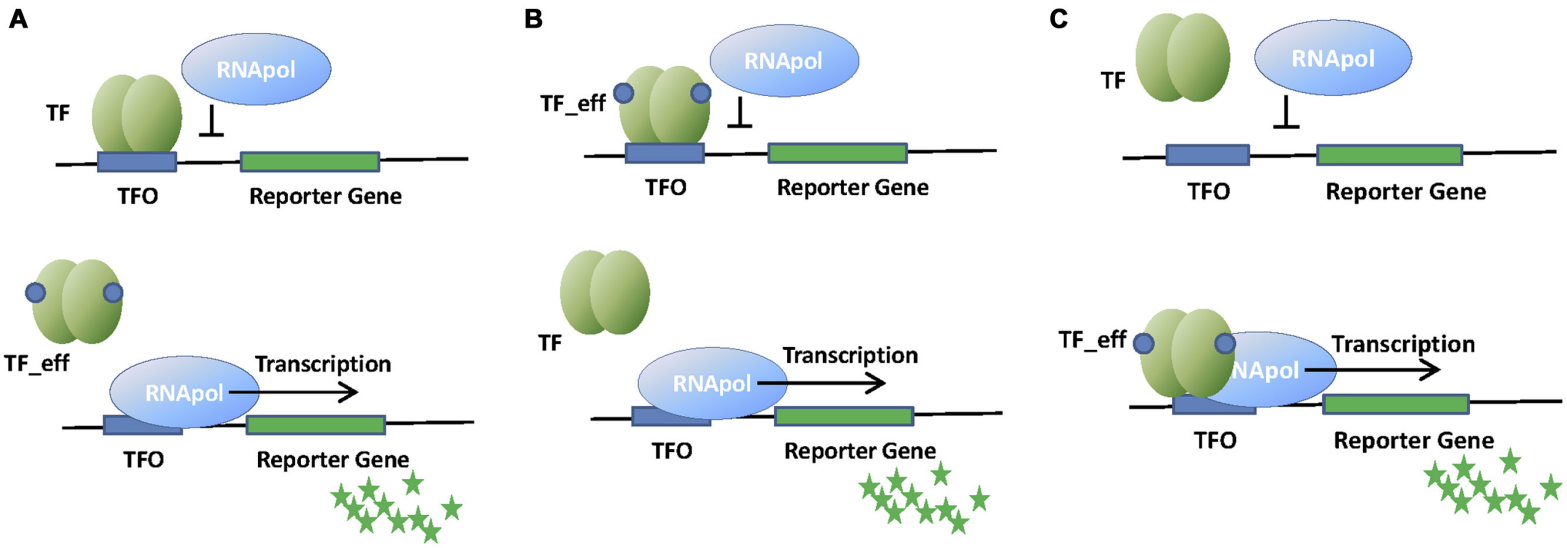
**Schematic representation of whole cell biosensors (WCBs) based on repressor **(A)**, aporepressor **(B)**, or activator **(C)** transcription factors (TFs).** Binding of TF to its operator (TFO) within the promoter region of the reporter gene affects RNApol activity and thus the signal associated to the reporter protein (green stars). The presence of the analyte (effector molecule) modifies the interaction of TF with TFO and changes the amount of reporter protein produced.

Transcription factors can act as transcriptional repressors or activators (**Figure 1**). When the effector is not present, transcriptional repressors are bound to their operator sites, which lay in the promoter region of the regulated operon (**Figure 1A**). When bound to DNA, transcriptional repressors block the association of the RNA polymerase (RNApol) to the promoter, or prevent its progression. Effector binding releases the repressor from its operator, allowing transcription of the operon. A variation of this regulation mode is used by aporepressors, which bind to DNA only if the effector (called corepressor) is present (**Figure 1B**).

Transcriptional activators bind to their operator sites by recruiting RNApol to the promoter, or inducing the formation of transcriptionally active RNApol-promoter open complexes when the effector is present (**Figure 1C**). Transcriptional activation is more complex than transcriptional repression, often requiring DNA bending and the establishment of specific contacts with RNApol α-subunit. We will describe these mechanisms in further detail when describing the relevant families of transcription activators.

## DNA Binding Domain

Bacteria have evolved a relatively short list of sequence-specific DBDs, commonly displaying one of three basic folds. Most frequently, DBDs contain the helix turn helix (HTH) motif. HTH motif is around 20 amino acids long and comprises two short alpha helices (7–9 amino acids long each). One is the DNA recognition helix while the second, perpendicular to the recognition helix, is the stabilizing helix (Brennan and Matthews, 1989). A short turn connects both helices, with a glycine usually conserved at the start of the turn. HTH DNA binding proteins bind to inverted repeat sequences separated by approximately one turn of helix. Thus, dimerization is required for full activity. Some HTH motifs contain additional alpha helices to stabilize the motif.

A variant of the HTH motif is the winged HTH motif (wHTH). In the canonical wHTH motif, a 3-helical bundle and a 3 strand β-sheet (wing) are arranged in the order: α1-β1-α2-α3-β2-β3 (Gajiwala and Burley, 2000). α2 and α3 form the regular HTH motif, α3 being the recognition helix involved in specific interactions with the major groove of the DNA.

A less frequent DNA binding motif is the ribbon helix helix (RHH) motif. It consists of a two-stranded anti-parallel β-ribbon followed by two α-helices. In RHH DNA binding proteins, two dimers contact each side of their cognate operator. DNA recognition is achieved by insertion of the β-ribbon into the major groove, whereas the two helices constitute most of the hydrophobic core and are involved in dimerization (Schreiter and Drennan, 2007). DNA specific contacts involve polar amino acids of their N-terminal β-sheets.

## Effector Binding Domain

While DBDs show a remarkable degree of conservation, EBDs are more variable, because of the chemical diversity of potential effectors. The role of the EBD is to bind the effector and transduce the activating/repressing signal. Signal transduction proceeds via conformational changes transmitted either to the DBD (causing its release from DNA in the case of transcriptional repressors) or to the RNApol (in the case of activators). The chemical specificity of EBD and its ability to produce a robust transcriptional signal is what qualifies a TF as a potential candidate for WCB development.

Structural and phylogenetic analyses identified TF families in prokaryotes. TF families usually share a common regulatory mechanism. Members of each family show sequence homology, with higher sequence conservation at the DBD. Yet, for many TF families, structural homology at the EBD can also be found. In most cases, members of a given TF family bind similar kinds of analytes, allowing a broad classification of TFs based on analyte specificity. There are, however, remarkable exceptions. As we will see, it is not rare to find cases where evolutionary exaptation produced TFs with different analyte specificity than most members of its family.

**FIGURE 2:**
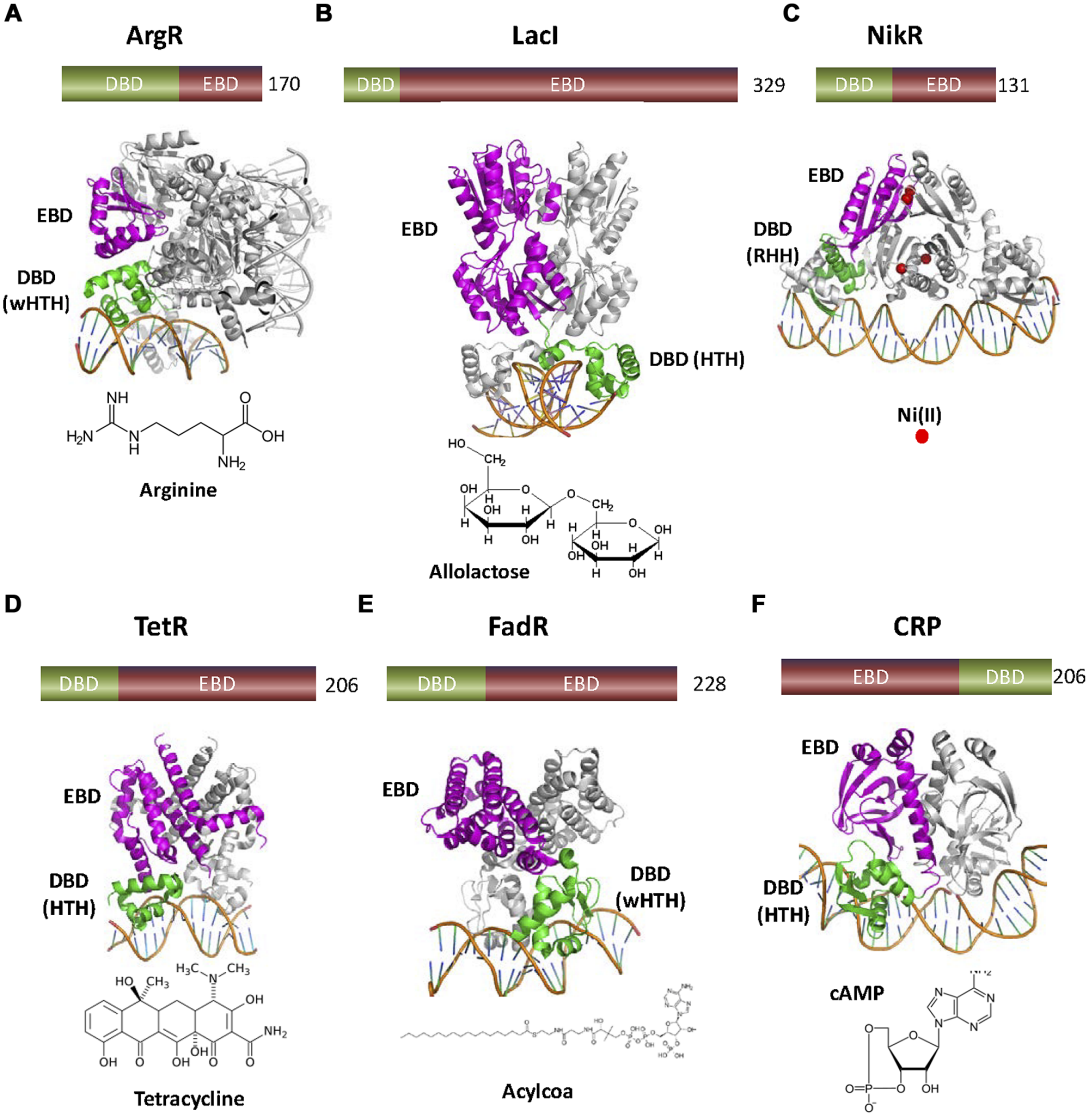
**Variety of one-component TFs found in nature. (A)** ArgR (3LAJ, Cherney et al., 2010) represses the transcription of the biosynthetic genes of the arginine operon. ArgR is a wHTH transcriptional aporepressor activated by the corepressor arginine, so that the regulated operon is not transcribed when the effector is present. **(B)** LacI (1EFA, Bell et al., 1998) inhibits expression of the *lac* operon. When the sugar allolactose is present, its binding to the C-terminal domain of dimeric LacI produces a conformational change that inhibits DNA binding by the N-terminal HTH. **(C)** NikR (2HZV, (Schreiter et al., 2006) is a ligand dependent aporepressor that only binds the operon when Ni^+2^ is bound. **(D)** TetR (1QPI, Orth et al., 2000) is a HTH repressor that senses the presence of the antibiotic tetracycline. **(E)** FadR (1HW2, Xu et al., 2001) contains an N-terminal wHTH motif connected to a C-terminal EBD similar to TetR family EDBs. However, FadR effectors are acyl-CoAs instead of antibiotics. **(F)** cAMP receptor protein or CRP (1CGP, Schultz et al., 1991) is a transcriptional activator. cAMP allows the binding of the CRP C-terminal HTH motif to the operator of several catabolic operons. There, CRP-cAMP interacts with RNApol, allowing the transcription of the corresponding operons. In this figure, as well as in the following figures, DBDs are colored in green and EBDs in magenta. The number of amino acids of each TF is also shown.

In general, TFs regulate the transcription of operons somehow related to their cognate analyte. For this reason, most TFs contain EBDs that recognize molecules involved in central metabolism. In nature, there are thousands of TFs with different mechanisms of transcriptional regulation (**Figure 1**), different effectors, different structural organization, or different DNA binding motifs (**Figure 2**).

Transcription factors like ArgR, LacI, or CRP constitute the hallmark of transcriptional regulation in bacteria. Most mechanisms that we will describe in the following sections where originally described for these TFs. Unfortunately, these TFs are of little interest for the purpose of WCB development. The role of biosensors is to provide the exquisite chemical specificity of biological components for the detection of compounds that are often hazardous, toxic, and/or contaminating. We will focus our attention in TF families that are used or could be used for the construction of WCBs to detect environmental contaminants such as aromatic compounds, antibiotics, or heavy metals. Depending on the recognized analyte, we grouped TFs in three main groups: TFs that respond to aromatic compounds, TFs for the detection of metal ions and TFs that respond to antibiotics.

## Detection of Aromatic Compounds

In the environment, aromatic hydrocarbons are a common source of toxicity. In fact, some aromatic compounds are endocrine disrupting chemicals (EDCs), toxic molecules associated with altered reproductive function, endocrine-related cancers (breast, endometrial, ovarian, prostate, testicular, and thyroid), abnormal growth patterns and neurobehavioral disorders (WHO, 2012). EDCs are found in various materials such as pesticides, additives, or contaminants in food, and personal care products emphasizing the need to detect their presence and concentration in these materials. Most of our knowledge on TFs that respond to aromatic compounds comes from the fields of biorremediation and natural pathways for biodegradation. Due to the toxicity of aromatic compounds, many bacterial species have evolved degradative pathways, often using these compounds as carbon sources for growth. Because exposure to these compounds is not constant, and the production of the enzymes required for degradation is metabolically expensive, the expression of degradative pathways is commonly regulated by the target compounds themselves (Ramos et al., 2009). Thus, many TFs employed by environmental bacteria for detoxification are ideal for the development of WCBs (Galvão and de Lorenzo, 2006).

Most TFs that act as biosensors for aromatic compounds were obtained from bacteria that thrive in polluted environments. Among them, *Pseudomonas putida* is the most widely used, due to its genetic tractability, culturability, and environmental versatility. Consequently, most data presented in this section comes from experiments performed in this, or closely related species.

Transcription factors for aromatic compound detection usually fall into three major families: XylS-AraC, NahR-LysR, and XylR-NtrC (**Table 1**). TFs from these families are generally transcriptional activators, although the specific mechanisms employed for transcription activation are different in each case.

**Table 1:** One-component transcription factor (TFs) with aromatic molecule effectors.

Effector	Regulator	Regulated system	PDB	Reference
**AraC/XylSF**				
Benzoate/*N*-toluate	XylS	Aromatic degradation genes Xyl		Inouye et al. (1981)
Benzoate/*N*-toluate	BenR	Benzoate degradation		Pérez-Pantoja et al. (2014)
**NtrC/XylRF**				
Toluene/*M*-xylene	XylR	Aromatic degradation genes Xyl		Devos et al. (2002)
Chlorinated phenols	DmpR	(Methyl)phenol degradation		Campos et al. (2004)
**LysRF**				
p-toluenesulfonate	TsaR	p-toluenesulfonate degradation genes tsaMBCD	3FXU	Monferrer et al. (2010)
Benzoate	BenM	Benzoate degradation	2F78	Ezezika et al. (2007)
Pentachlorophenol, trichlorophenol	PcpR	Polychlorophenol degradation	4RPN, 4RPO	Hayes et al. (2014)
Salicylate	DntR	2,4-dinitrotoluene (DNT) degradation	2Y7K	Devesse et al. (2011)

## XylS-AraC Family

**FIGURE 3:**
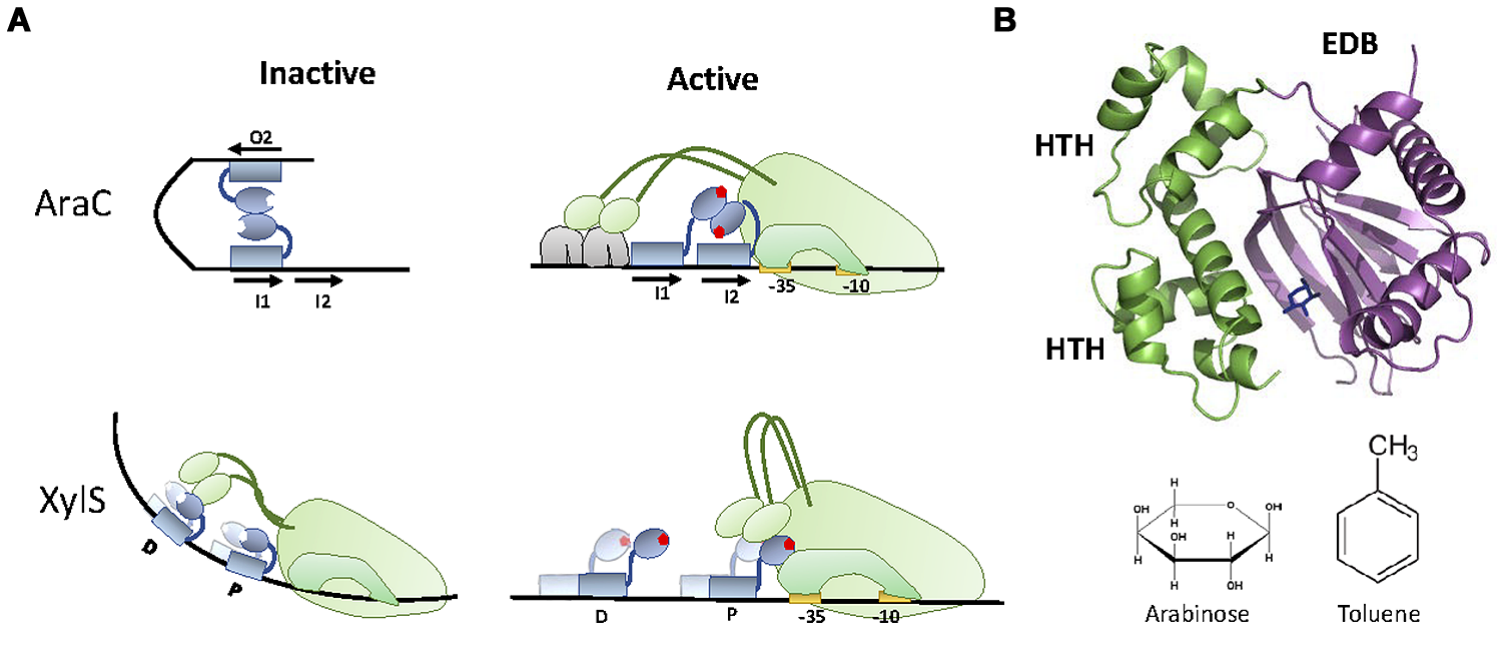
**XylS-AraC family. (A)** Activation mechanism. In the repression state, AraC bound to I1 and O2 at the *araBAD* promoter forms a DNA loop that prevents transcription initiation. When arabinose is present, each of the AraC monomers bind adjacent operators (I1 and I2), releasing DNA bending and promoting the recruitment of RNApol and subsequent transcriptional activation. XylS activation mechanism is different, being RNApol repressed by apo-XylS bound to operator D and activated by tolune-XylS bound to operator P. **(B)** XylS-AraC structure. The structure of XylS homolog ToxT (3GBG, Lowden et al., 2010) is shown together with the location of arabinose in the EBD of AraC (2ARC). The two HTH motifs present in the DBD are highlighted. AraC (arabinose) and XylS (toluene) effectors are shown.

Many TFs that respond to aromatic compounds belong to the AraC superfamily of transcriptional regulators. AraC is a transcriptional activator that drives the expression of the arabinose operon in *Escherichia coli*, and the prototype of a TF family that contains more than 10,000 homologs (Yang et al., 2011). Extensive studies in the last 40 years have unveiled AraC mechanism of action in great detail. AraC is a dual TF, acting as a transcriptional repressor in its *apo* form, and as a transcriptional activator when bound to arabinose. The canonical AraC “light switch” mechanism of transcriptional control involves two states. In its *apo* form, AraC forms a dimer and binds to the *araBAD* promoter via two distant operators (I1 and O2; **Figure 3**). In this state, DNA is bent forming a loop that prevents transcription initiation. Binding of arabinose to AraC produces an allosteric change in the protein. This allosteric change forces the AraC to bind the adjacent operators I1 and I2, relaxing the DNA loop. The relaxed state of the promoter allows the recruitment of the RNApol by the general regulator CRP, thus promoting transcription. This activation mechanism is similar in other members of the AraC family.

Members of the AraC superfamily contain a C-terminal DBD, and an N-terminal EBD (which is also responsible for protein dimerization; Bustos and Schleif, 1993). The DBD contains two HTH motifs (**Figure 3B**), although only the second one (N-terminal) makes specific contacts with the operator sequence (Gallegos et al., 1997). This DBD is conserved among all members of the AraC superfamily. In contrast, the EBD has suffered a remarkable degree of evolutionary exaptation, producing AraC-like TFs responding to a wide range of different analytes. For WCBs, the most interesting AraC-like TFs belong to a specific sub-family, represented by XylS, a TF from *P. putida* mt-2.

XylS regulates a degradative pathway present in plasmid pWW0 that allows *P. putida* mt-2 to degrade toluene and *m*-xylene. This route is divided in two operons: the upper (ortho) and the lower (meta) operons. XylS is responsible for the transcriptional control of the lower part (the upper part is controlled by XylR, which will be described in the next section). XylS activates transcription in response to benzoate and *m*-toluate, intermediate metabolites generated by the upper part of the metabolic pathway, and substrates for the enzymes encoded in the meta operon. XylS mechanism of action is slightly different from AraC, since the over expression of XylS can trigger transcriptional activation independently of the inducer (Ramos et al., 1987). Also, the mechanism of activation of XylS upon binding to its inducer is not identical to AraC. In AraC, the apo form of the protein suffers from intermolecular repression (the TF is kept in its inactive form by specific contacts between monomers), while XylS suffers from intramolecular repression (Domínguez-Cuevas et al., 2008).

Regarding analyte specificity, although XylS-AraC family members exhibit substantial sequence identity at the EBD, they recognize widely different analytes. Some, like BenR, exhibit analyte specificities similar to XylS (Pérez-Pantoja et al., 2014). Others participate in the regulation of virulence determinants in different microbial pathogens. These TFs recognize molecules as varied as bicarbonate (RegA from *Citrobacter rodentium*), cellobiose (TxtR from *Streptomyces scabies*), urea (UreR from *Proteus mirabilis*), and bile salts (ToxT from *Vibrio cholerae*; Yang et al., 2011). Unfortunately the structural basis for this remarkable analyte diversity is poorly understood. Although the crystalline structures of AraC DBD and EBD domains have been obtained (Soisson et al., 1997; Rodgers and Schleif, 2009), the lack of structural information for XylS-like TFs makes the molecular basis of substrate recognition obscure. Even though, PHYRE (Kelley and Sternberg, 2009) predicts XylS structure to be similar to ToxT (3GBG; Lowden et al., 2010), indicating that slight structural changes may result in radical alterations of effector specificity. Despite the lack of structural information for rational engineering, mutagenesis studies have been successful in generating XylS molecules with altered inducer specificity (Michan et al., 1992). Among them, XylS2 mutant stands out for its ability to detect a wide range of inducers, including salicylate (Ramos et al., 1986).

## XylR-NtrC Like Transcriptional Regulators

**FIGURE 4:**
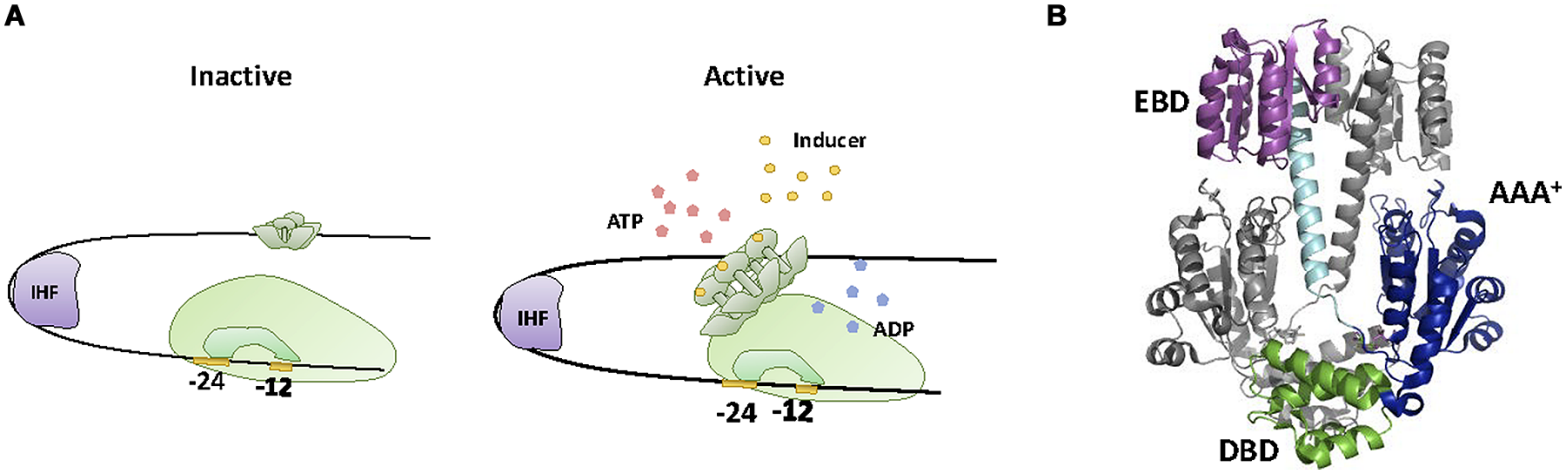
**XylR-NtrC family. (A)** Activation mechanism. NtrC effector produces NtrC hexamerization to an ATPase active form, in a way that allows NtrC to make specific contacts with the σ^54^-RNApol holoenzyme and thus remodeling the transcription complex from its closed configuration into an open transcriptionally active form. Activation requires the formation of a DNA loop, aided by IHF. **(B)** NtrC structure. The crystal structure of the inactive dimer of NtrC1 (1NY5, Lee et al., 2003) is shown. DBD, EDB and AAA^+^ domains as well as the alpha helix that connects EDB with AAA^+^ are highlighted.

A second group of TFs involved in sensing and degradation of aromatic compounds is the NtrC family. Members of this family are usually involved in the expression of adaptive genes for harsh environmental conditions (Kustu et al., 1989), promoting transcription via σ^54^ mediated activation of RNApol (Hirschman et al., 1985). σ^54^ dependent promoters display a -12/-24 architecture that renders the formation of the open complex thermodynamically unfavorable (Hunt and Magasanik, 1985; Thöny and Hennecke, 1989). NtrC-like TFs activate transcription by providing the energy required for the formation of the open complex (Weiss et al., 1991). For this purpose, NtrC-like proteins contain an AAA+ ATPase motif that constitutes a distinct feature of this TF family (De Carlo et al., 2006). Another feature of NtrC-like regulators is the unusual location of their cognate DNA binding sites. These sites, named upstream activator sequences (UASs), are situated up to 200 bp upstream the transcriptional start (Pérez-Martín et al., 1994), resembling eukaryotic enhancers. NtrC activation involves protein multimerization into an ATPAse active form [most commonly into a hexamer, but occasionally into a heptamer (De Carlo et al., 2006; Bush and Dixon, 2012)]. Since UASs are located far up the transcriptional start, activation requires the formation of a DNA loop that allows NtrC to make specific contacts with the σ^54^-RNApol holoenzyme (**Figure 4A**). DNA looping is aided either by DNA intrinsic curvature or by other DNA-binding proteins such as IHF (Bush and Dixon, 2012). Once the loop has been formed and contacts between the TF and the σ^54^-RNApol holoenzyme established, the energy produced by ATP hydrolysis is invested in remodeling the transcription complex from its closed configuration into an open, transcriptionally active form (Rappas et al., 2006).

Structurally, NtrC family TFs (**Figure 4B**) contain a conserved and a variable region (Bush and Dixon, 2012). The conserved region contains an N-terminal domain that bears the HTH motif responsible for UAS binding (DBD), and a large, central domain that contains the AAA+ fold for ATPase activity (De Carlo et al., 2006). The variable region contains the structural determinants that control oligomerization and ATPase activity. The entire NtrC-like family shows a remarkable degree of variability in this respect, with about 50% of its members containing a regulatory domain for the specific interaction with another protein (two-component systems and protein–protein interactions) while the other 50% exhibit a sensing motif that activates transcription upon binding a small ligand (Bush and Dixon, 2012). NtrC-like proteins involved in aromatic compound sensing and degradation are among the latter, containing a C-terminus region that binds to the effector and regulates oligomerization and ATPase activity (North et al., 1993). For these purposes, the C-terminus of the protein is divided in two domains. The A domain is involved in ligand binding, while the B domain acts as a hinge that brings together the A domain and the AAA+ fold located at the central part of the protein. In this conformation, the A domain suppresses protein oligomerization and ATPase activity. Upon ligand binding, intra-domain repression is relieved, resulting in protein oligomerization and ATPase activation (Pérez-Martín and De Lorenzo, 1995).

Several NtrC-like TFs respond to aromatic compounds, but the model system for this entire group is XylR, the transcriptional regulator of the upper operon for toluene and *m*-xylene degradation from plasmid pWW0. XylR responds to *m*-xylene and toluene, substrates for pWW0 upper pathway, but also to a surprising variety of structural analogs (reviewed in Galvão and de Lorenzo, 2006). This versatility was exploited to generate biosensors for BTEX (benzene, toluene, ethylbenzene, and xylene, a common source of contamination resulting from the oil industry) not only in the lab (Mi Na Kim, 2005) but also *in situ* (de las Heras and de Lorenzo, 2011b). XylR was also engineered to detect nitrotoluenes, a promising approach for the bio-detection of landmines (Garmendia et al., 2008; de Las Heras and de Lorenzo, 2011a). Recently, novel XylR variants that allow the implementation of simple Boolean logic operations were generated (Calles and de Lorenzo, 2013). Similarly, network engineering allowed the generation of sensing circuits based on XylR that display enhanced analyte specificity, overcoming the natural promiscuity of XylR (de Las Heras et al., 2012). These advances turned XylR into a most attractive TF for biosensor development.

Another NtrC-like TF that was successfully turned into a biosensor is DmpR (Campos et al., 2004; Gupta et al., 2012). DmpR is a transcriptional regulator from plasmid pVI150 that confers *P. putida* the ability to grow on phenols or methyl-phenols (Shingler et al., 1989). DmpR is 65% identical to XylR at the amino acid level, thus the protein structure and mechanism of action are likely to be similar. Yet XylR and DmpR show different analyte specificities (Galvão and de Lorenzo, 2006) protein engineering by domain shuﬄing identified the amino acids involved in this differential specificity (Skärfstad et al., 2000).

## LysR-Type Transcriptional Regulators

**FIGURE 5:**
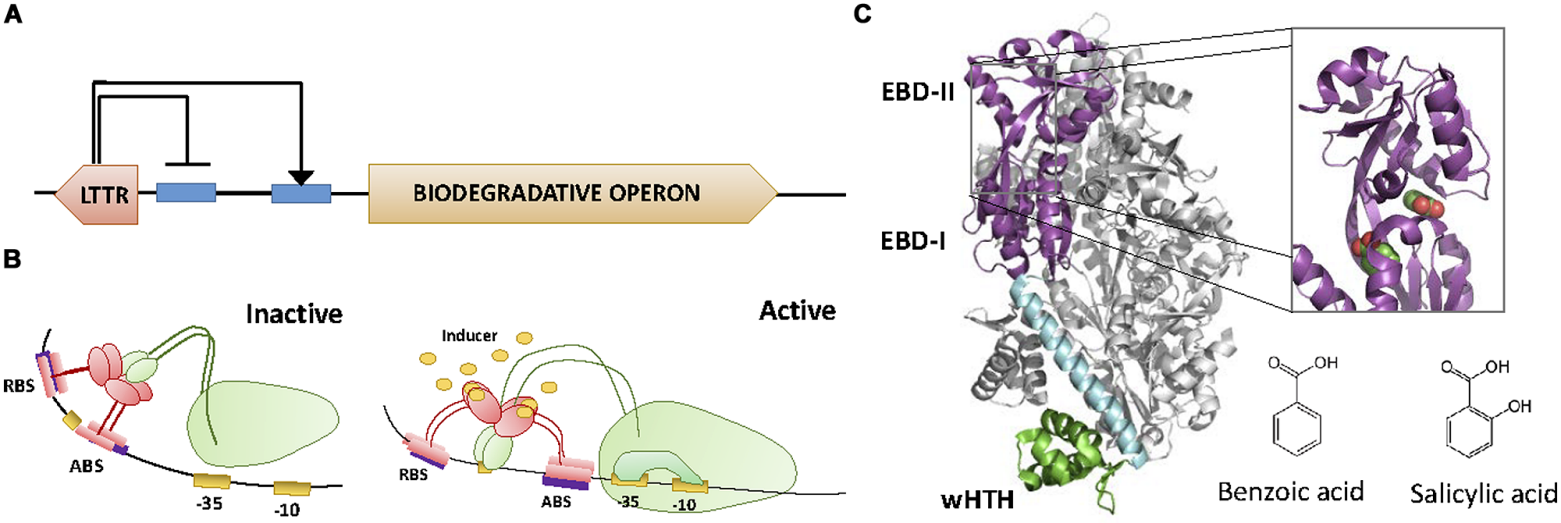
**LysR family. (A)** Architecture of the LTTR regulator and the regulated operon. LTTRs repress their own synthesis, while activating the transcription of the biodegradative operon. **(B)** Activation mechanism. Each of the dimers of the tetramer LTTRs binds to the RBS and the activating binding site (ABS), respectively. This causes the DNA to bend, preventing the access of the α-CTD of the RNApol to the promoter. Upon binding to the effector, the protein slides from ABS to ABS″. This movement directs the α-CTD of RNApol to contact DNA promoting transcription. **(C)** LTTR structure. The crystal structure of the LTTR CnbR (1IZ1, Muraoka et al., 2003) is shown. The inset shows the location of two benzoic acid molecules within BenM (2F78, Ezezika et al., 2007). The molecular structure of effectors benzoic acid and salicylic acid is also shown.

The LysR family of transcriptional regulators (LTTRs) constitutes the most abundant family of TFs found in bacteria (Pareja et al., 2006). Its members are usually around 300 amino acids long. They display a basic structure consisting of an N-terminal DBD containing an HTH fold, and a C-terminal EBD (Maddocks and Oyston, 2008). LTTRs typically behave as dual transcriptional repressors/activators (Maddocks and Oyston, 2008). Divergent transcription of the regulator and the regulated operon is a common theme among these TFs. LTTRs generally exert a repressive action on their own synthesis (thus engaging in negative feedback loops), while activating transcription of the metabolic pathway upon binding to their cognate effectors (**Figure 5A**). Transcriptional regulation by LTTRs usually involves two operators, known as the regulatory binding site (RBS) and activating binding site (ABS). In the case of LTTRs involved in aromatic compound metabolism, the RBS is located between the -50 and the -80 position of the promoter, while the ABS is located closer to the transcriptional start (ranging from -50 to -35). The mechanism of action for LTTRs involves a protein tetramer binding simultaneously to both RBS and ABS sites, with one dimer binding RBS and another binding ABS (**Figure 5B**). This causes the DNA to bend, preventing access of the α-CTD of RNApol to a specific DNA region in the promoter (called UP; Tropel and van der Meer, 2004). Upon binding the effector, the protein slides from the ABS site to an upstream position (ABS″). This movement induces relaxation of DNA bending and directs the α-CTD of RNApol to contact the UP region of the DNA (Devesse et al., 2011). This is the active configuration that promotes transcription.

Several LTTRs are known to respond to aromatic compounds, but unlike XylS and XylR families, there is not a clear prototype for the entire group. Instead, LTTRs involved in the degradation of aromatic compounds can be broadly divided in distinct subgroups according to their effectors (which correlates with sequence similarity at the C-terminal EBD). Among them, two subgroups deserve specific attention. The first comprises LTTRs that respond to cis,cis-muconate or its chlorinated derivative 2-chloro-cis,cis-muconate, intermediates in the degradation pathway of catechol and chlorocatecol, respectively. Main representatives of this subgroup include CnbR, from *Ralstonia*, ClcR and CatR from *Pseudomonas*, and BenM from *Acinetobacter*. Crystal structures of CnbR (1IZ1, Muraoka et al., 2003) and BenM (2F78, Ezezika et al., 2007) shed light on the molecular basis of effector recognition for this group of closely related LTTRs (**Figure 5C**). CnbR and BenM display 28% amino acid sequence identity and significant structural similarity. Their C-terminus contains two motifs, EBD-I and EBD-II, connected by a hinge (Schell, 1993; Muraoka et al., 2003; Ruangprasert et al., 2010). This arrangement is similar to the prototypic periplasmic binding protein (PBP) fold, in which two globular β/α domains, separated by a hinge, form a clamp that binds a small molecule (Ruangprasert et al., 2010). In the case of CnbR and BenM, the effector binding clamp is formed between EBD-I and EBD-II (Muraoka et al., 2003; Ruangprasert et al., 2010). Characteristic of these proteins is the formation of an asymmetric tetramer (Bundy et al., 2002; Muraoka et al., 2003), resulting from the association of two dimers in different conformations (Muraoka et al., 2003; Ruangprasert et al., 2010). One protein from this group, BenM, shows the distinct property of having two inducers that act synergistically. BenM is induced by cis,cis-muconate and benzoate (Bundy et al., 2002; Clark et al., 2002). The synergistic effect of both inducers is possible because BenM presents two distinct binding sites, one for cis,cis-muconate (present at the clamp between EBD-I and EBD-II domains) and a second one for benzoate (Craven et al., 2009). This dual regulatory input is unique among TFs that respond to aromatic compounds.

A second subgroup of LTTRs involved in the degradation of aromatic compounds is represented by NahR from *Pseudomonas* and DntR from *Burkholderia*. These two regulators exhibit a 40.5% identity in their amino acid sequence and they both respond to the inducer salicylate, an intermediate metabolite in the degradation pathway of naphthalene. Although NahR was successfully implemented in functional salicylate biosensors (Werlen et al., 2004; Shin, 2010; Xue et al., 2014), more detailed information about the molecular mechanism of DntR exists in the literature. Among TFs that control degradative pathways of aromatic compounds, DntR is exceptional due to its unusual effector specificity. As mentioned above, DntR responds to salicylate. Yet DntR is associated with a biodegradative pathway for 2,4-dinitrotoluene (DNT), for which salicylate is not an intermediate metabolite. Actually, DntR is unresponsive to DNT or any of its intermediate catabolites. Thus DntR was considered a substrate-blind regulator (de Las Heras et al., 2011). To explain this bizarre situation, de Las Heras et al. (2011) noted that DNT is a xenobiotic compound, not found in the environment. Thus, it is likely that the DNT pathway regulated by DntR represents a recent evolutionary innovation, involving the adaptation of naphthalene degradation genes to mineralization of DNT, with a TF that is still poorly adapted (de Las Heras et al., 2011). Besides representing a beautiful example on how xenobiotic stress drives the evolution of environmental bacteria, the abnormal effector profile of DntR serves also as a cautionary tale for the development of biosensors. It indicates that one cannot take for granted that the transcriptional regulator of a catabolic pathway is going to respond to the substrate or any of the intermediates of the pathway. This is especially likely if the substrate is a xenobiotic compound that has been introduced in the environment in recent times (at the evolutionary scale). Regarding the structural basis of DntR activation by salycilate, crystallographic data indicated that its structure is similar to that of other LTTRs, with a C-terminus consisting of two subdomains linked by a hinge (Devesse et al., 2011). As is the case in BenM and CnbR, the effector binding pocket is located in the interface between these two subdomains (Devesse et al., 2011). Interestingly, DntR shows a second pocket for salicylate binding, which resembles the double binding pocket present in BenM. Although the physiological relevance of the second binding site is not clear (Devesse et al., 2011), it is tempting to speculate that it might represent an intermediate step in the adaptation of DntR to the recognition of DNT or any of its intermediate catabolites. Experimental evolution resulted in DntR variants that show a 10-fold increase in DNT sensitivity, but these mutants also exhibited higher sensitivity for salicylate (Lönneborg et al., 2012).

## Detection of Metal Ions

Metal detection is a fundamental goal of WCB development. Some metals, such as Cu, Fe, K, Mg, or Mn are essential nutrients, while others (Ag, Al, Cd, Au, Pb, Hg) serve no known biological function (Bruins et al., 2000). Nevertheless, most metals have toxic effects in biological reactions at high concentrations, regardless of being essential or non-essential. Toxicity is the main reason behind the development of metal biosensors. WCBs were successfully constructed to detect Hg(II) in environmental samples (Bontidean et al., 2004; Priyadarshi et al., 2012). Cadmium is detected using WCBs containing GFP-metallothionein (Amaro et al., 2014) and WCBs with an engineered modular genetic AND logic gate are able to detect As(III), Hg(II), Cu(II), and Zn(II) and distinguish between them (Wang et al., 2013). The economic value of some metals also justified the development of biosensors for mining purposes (Cerminati et al., 2011). Based on their structural homology and analyte specificity, five main families of prokaryotic metal sensing transcriptional regulators can be defined (Pennella and Giedroc, 2005).

## MerR Family

The MerR family comprises a group of TFs that generally respond to transition metals and act as transcriptional activators. The main representative of this family is MerR, a TF present in Gram+ and Gram- bacteria, which binds to mercury and regulates the expression of a mercury resistance operon (Barrineau et al., 1985).

MerR is a transcriptional activator that binds to a specific operator located between the -35 and -10 elements of a σ^70^ promoter. In MerR-responding promoters the space between the -35 and -10 elements is 19–20 bp long, greater than the optimal 17 ± 1 bases required for adequate transcriptional activity (Ross et al., 1989). This elongation causes limited transcriptional activity. Moreover, the apo form of MerR binds to its operator as a dimer, blocking transcription initiation, thus acting as a transcriptional repressor. Upon binding to its effector, MerR causes a slight bend on the operator DNA, approaching the -10 and -35 sequences, thus favoring the association of RNApol (Ansari et al., 1995) and promoting the formation of the transcriptional open complex (Brown et al., 2003).

**FIGURE 6:**
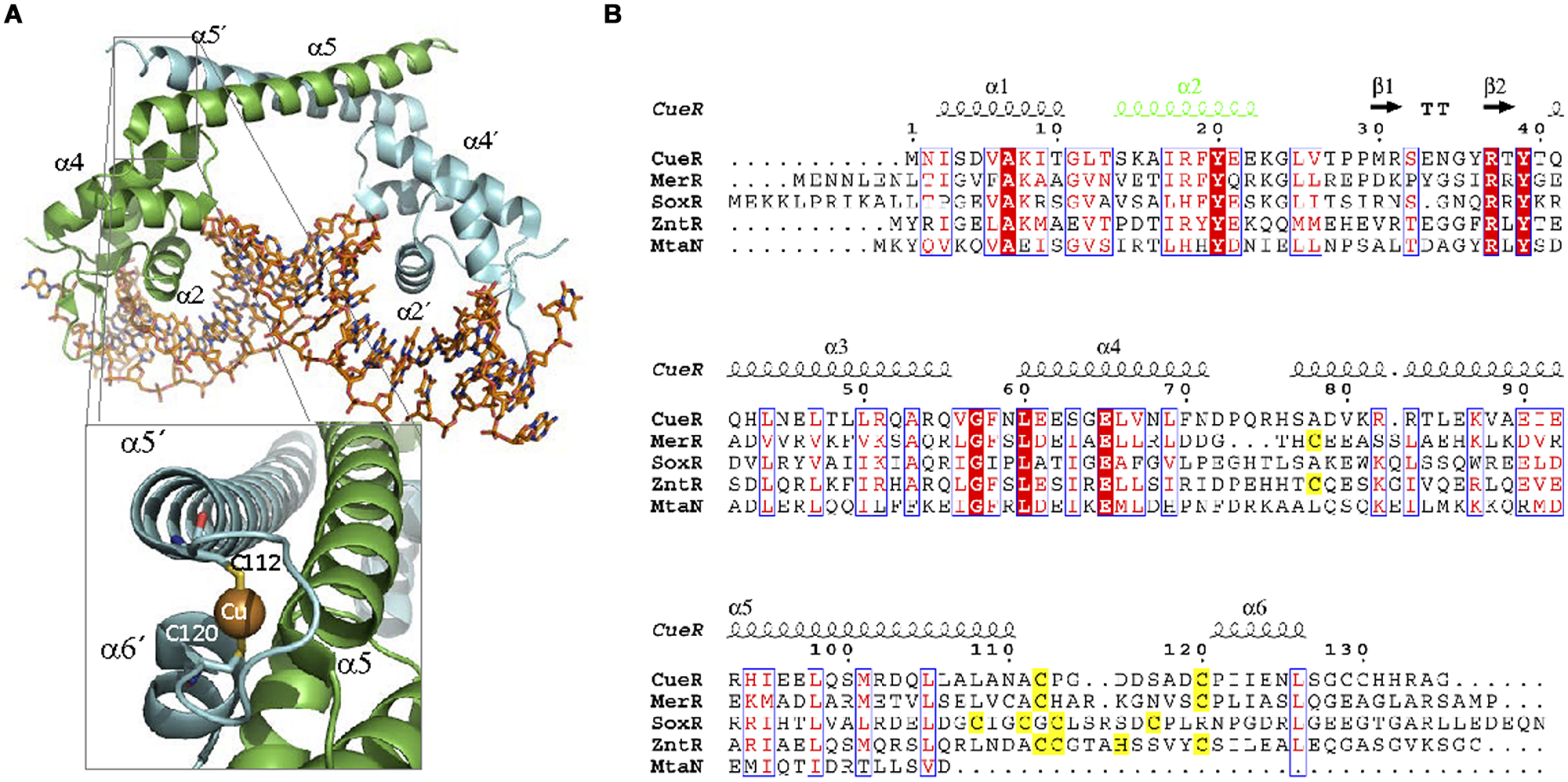
**MerR family. (A)** MerR structure. The structure of the MerR member MtaN bound to DNA (1R8D, Newberry and Brennan, 2004) illustrates MerR family binding to its operator. MtaN monomers are shown in green and blue, respectively, while DNA is shown in orange. The inset shows the two cysteines of CueR (1Q05, Changela et al., 2003) involved in Cu (I) coordination. **(B)** MerR family alignment. The alignment of representative members of MerR family is shown. Residues involved in metal coordination are highlighted in yellow. DNA binding α2 is shown in green.

Structurally, MerR proteins are characterized by three distinct domains: an N-terminal DBD domain, a central linker, and a C-terminal EBD (**Figure 6A**). The DBD contains a HTH motif formed by the first 44 amino acids of the protein (Brocklehurst et al., 1999). Helices α1 and α2 form the DNA binding HTH motif while the next two helices (α3 and α4) comprise a coupling region that communicates occupancy of the EBD to the DBD (Guo et al., 2010). These helices are followed by a coiled coil region (α5) that is involved in protein dimerization (amino acids 80–128; Zeng et al., 1998). The EBD contains a metal binding pocket formed by three cysteines (**Figure 6**). Two Cys come from one monomer (Cys117 and Cys126 in *E. coli* MerR) and one Cys comes from the other monomer (Cys82′ in *E. coli* MerR; Utschig et al., 1995). This site is essential for metal binding. In MerR the Cys center binds to Hg(II), but other members of the family can bind toxic metals like Br (BmrR) and Pb (PbrR), or micronutrients like Cu (CueR) and Zn (ZntR; **Table 2**). Although metal binding is always exerted through the Cys coordination, the number of Cys residues involved is not conserved. In some cases MerR regulators can bind to different ions using a distinct subset of these Cys residues. For example, ZntR can be activated by Zn(II), which binds to five Cys residues. However, the same protein can be activated by Pb(II) and Cd(II), which require coordination with only four Cys residues.

**Table 2:** One-component TFs with metal ion effectors.

Effector	Regulator	Regulated system	PDB	Reference
**MerRF**				
Hg (II)	MerR	Mercury detoxification		
Cu (I), Ag (I), Au (I)	CueR	Copper-exporting ATPase, CopA	1Q05 (Cu), 1Q06 (Ag), 1Q07 (Au)	Changela et al. (2003)
Zn (II)	ZntR	ZntA Zn(II)/Cd(II) export gene	1Q08	Changela et al. (2003)
**ArsRF**				
As (III)	ArsR	Arsenic resistance		Busenlehner et al. (2003)
Zn (II)	SmtB	Cellular resistance to excess zinc	1R23	Eicken et al. (2003)
Zn (II)	CzrA	Zinc resistance	1R1V	Eicken et al. (2003)
Ni(II), Co(II)	NmtR	ATPase metal eﬄux pump	2LKP	Lee et al. (2012)
Cd (II), Pb(II), Zn(II)	CadC	Heavy-metal eﬄux pump CadA	1U2W	Ye et al. (2005)
**DtxRF**				
Fe (II), Ni(II)	DtxR	Diphteria toxin regulation	1DDN	White et al. (1998)
Fe (II), Co(II), Ni(II)	IdeR	Iron uptake	2ISY	Wisedchaisri et al. (2007)
Mn(II), Cd(II), Zn(II)	ScaR	Manganese uptake	3HRT (Cd) 3HRU (Zn)	Stoll et al. (2009)
**FurF**				
Fe (II)	Fur	Iron uptake	2W57	Sheikh and Taylor (2009)
Zn (II)	Zur	Zinc-uptake	4MTE	Gilston et al. (2014)
Ni (II)	Nur	Nickel homeostasis and anti-oxidative response	3EYY	An et al. (2009)
**NikRF**				
Ni (II)	NikR	Nickel ABC-type transporter	2HZV	Schreiter et al. (2006)

This Cys center characteristic of MerR regulators is primarily suited for metal binding. Yet evolutionary exaptation has generated MerR regulators where these Cys residues constitutively bind to a metal ion to respond to physichochemical signals. The most paradigmatic example of these type of MerR regulators is the oxidative stress sensor SoxR (Amábile-Cuevas and Demple, 1991). Binding to four metal ions [2Fe-2S], SoxR is able to sense superoxide concentrations (Ding et al., 1996). Other MerR-like regulators are able to sense antibiotics (TipA, Holmes et al., 1993), or even light (LitR, Takano et al., 2011).

The modular structure of MerR-like regulators allowed the construction of chimeric TFs. The proof of principle for this approach is the hybrid MerR-ZntR constructed by Brocklehurst et al. (1999). It comprises the N-terminal region of Tn501 MerR (44 amino acids) and 103 amino acids from the C-terminal region of ZntR. This hybrid MerR-ZntR senses Zn(II) and was expected to regulate the expression of MerR-responding promoters. However, although the hybrid TF is able to bind to its cognate operator, it is not able to activate transcription from this promoter. The hybrid MerR-ZntR TF requires a chimeric promoter that includes the 20 bp spacer of ZntR-responding promoters (Brocklehurst et al., 1999). These results indicate that the conformational changes that promote DNA binding reside in the C-terminal domain, and not in the N-terminal DBD.

## ArsR Family

The SmtB/ArsR TF family is also involved in metal sensing. They control operons involved in protection against toxic metals, thus constituting an interesting source of TF for metal biosensing. Their mechanism of action is relatively simple. They are repressors that bind their cognate promoters, recognizing an operator located near or overlapping the transcriptional start site. Upon binding the inducer metal ion, ArsR-like TFs are released from the promoter, thus allowing transcription (Eicken et al., 2003). They occur in Gram positive, Gram negative bacteria and also in *archaea* (Itou et al., 2008). Important members of this family are the As sensor ArsR of *E. coli* (P15905, Busenlehner et al., 2003), the Cd sensor CadC of *Staphylococcus aureus* (1U2W, Ye et al., 2005), the Zn sensors SmtB of *S. elongatu*s (1R23, Eicken et al., 2003) and CzrA of *S. aureus* (1R1V, Eicken et al., 2003) and the Ni sensor NmtR of *M. tuberculosis* (2LKP, Lee et al., 2012), **Table 2**.

**FIGURE 7:**
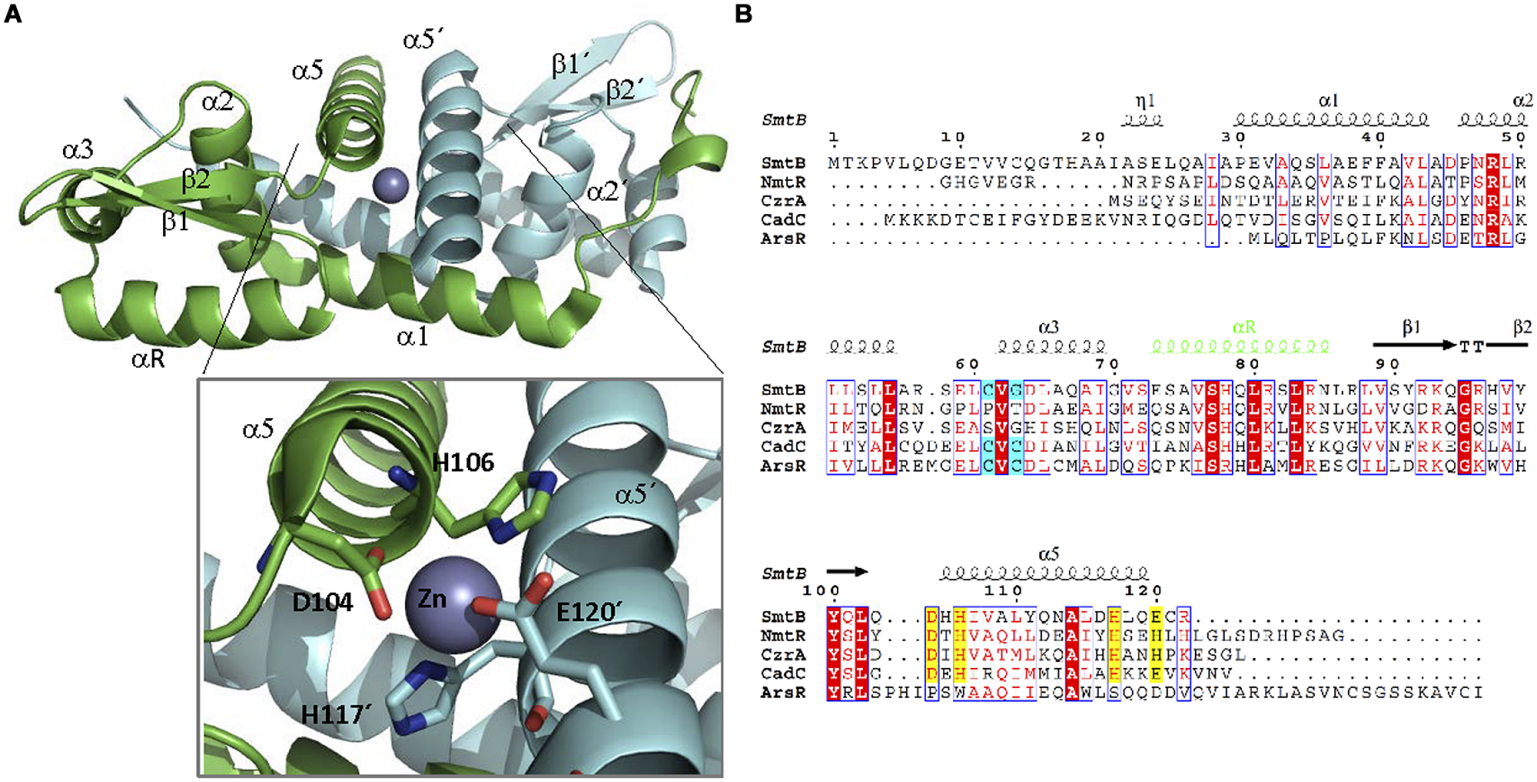
**ArsR family. (A)** ArsRF structure. The crystal structure of the dimer SmtB bound to Zn (II) (1R23, Muraoka et al., 2003) is shown. SmtB monomers are shown in green and blue, respectively. The inset shows the residues within α5 involved in metal binding. **(B)** ArsR family alignment. The alignment of ArsR members of **Table 2** is shown. Residues of α3 or α5 involved in metal binding are highlighted in blue and yellow, respectively. DNA binding αR is shown in green.

Structurally, ArsR family members share a similar folding (Itou et al., 2008; Zhao et al., 2010; Mukherjee et al., 2014). They form dimers (Busenlehner et al., 2003) with each monomer exhibiting a α1-α2-α3-αR-β1-β2-α5 organization (**Figure 7A**, Cook et al., 1998). The dimerization interface is constituted by α1 and α5 helices. The DBD presents a wHTH motif formed by α3 and αR, which is the most conserved region of the protein family (Busenlehner et al., 2003). The effector is bound by two metal coordination sites. The first is located in helix α3, adjacent to the DBD, while the second is found in α5, at the dimerization interface (Shi et al., 1996; Turner et al., 1996; VanZile et al., 2002; Busenlehner et al., 2003). As in the case of MerR-like regulators, metal coordination is exerted by a series of Cys residues. Interestingly, not all members of the ArsR family present both metal coordination sites (**Figure 7B**): ArsR and *Listeria monocytogenes* CadC present only α3, CzrA and NmtR present only α5 while CadCs, SmtB, and ZiaR present both (Busenlehner et al., 2003). For those ArsR-like regulators that exhibit two metal coordination sites, only one seems to be involved in signal transduction. SmtB responds through its α5 metal binding motif, while CadC does it through the α3N site (Busenlehner et al., 2003).

Biosensors built upon ArsR constitute some of the most successful approaches for the practical use of WCBs. Arsenic contamination in drinking water is a considerable public health problem in several parts of the world (Tchounwou et al., 1999). Because arsenic determination by chemical methods is unaffordable in certain areas, WCBs constitute an appealing low-cost alternative. Field studies have demonstrated the applicability of ArsR biosensing in rural areas (Trang et al., 2005; Siegfried et al., 2012).

## DtxR, Fur, and NikR Families

Besides MerR and ArsR families, which comprise TFs associated to metal resistance and detoxification, other TFs are able to bind metals and elicit transcriptional responses. Transition metals are key cofactors for enzymatic catalysis and essential components of many cellular proteins. However, they cannot be accumulated in the cell, since any excess may result in the formation of reactive oxygen species. Thus, bacteria keep a delicate balance of metal homeostasis. Three families of transcriptional repressors, DtxR, Fur, and NikR, are key in this process. These regulators not only have a homeostatic role. Metal deprivation is a common way of fighting infections employed by eukaryotic organisms. Many pathogens have evolved regulatory circuits that link the expression of virulence factors with metal distress (Kehl-Fie and Skaar, 2010). These sensing capabilities make Dtx, Fur, and NikR-like TFs appealing candidates for the development of metal WCBs.

**FIGURE 8:**
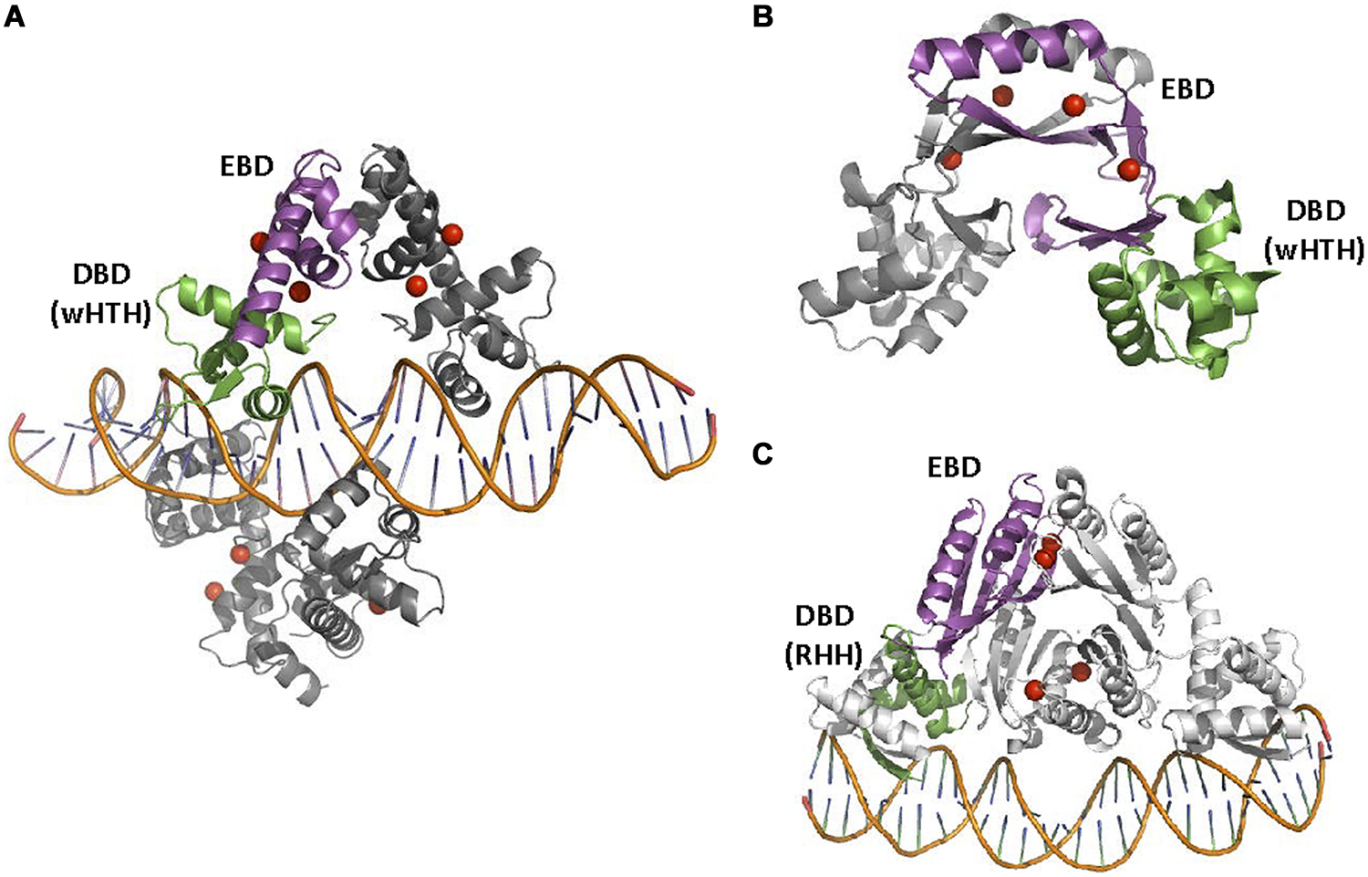
**DtxR, Nur, and NikR families. (A)** DtxRF structure. Two DtxR dimers are shown, bound to DtxR operator (1DDN, White et al., 1998). **(B)** FurF structure. The Ferric uptake regulator (Fur) is bound as a dimer to its operator (2W57, Sheikh and Taylor, 2009). **(C)** NikRF structure. NikR recognizes its operator sequence by a RHH DBD (2HZV, Schreiter et al., 2006). The location of Ni(II) atoms in DtxR and NikRA and Zn(II) atoms in Fur is shown by red spheres.

DtxR is the prototype of a family of transcriptional aporepressors involved in metal homeostasis and metal-dependent virulence regulation (Andrews et al., 2003). Members of this family are able to detect iron (DtxR, IdeR, SirR; Hill et al., 1998) and manganese (ScaR, MntR, and TroR; Stoll et al., 2009; Liu et al., 2013) although, like most metal-dependent TFs, they display effector promiscuity, binding other cations with lower affinity (Pennella and Giedroc, 2005). Structurally, DtxR-like repressors present two conserved domains and one variable domain (Stoll et al., 2009). The N-terminal DBD contains a wHTH motif that is conserved among all members of the family, along with a central domain involved in protein dimerization (**Figure 8A**). SirR, ScaR, and IdeR contain also a FeoA-like C-terminal domain of uncertain function, not present in other members of the family. Domain variability is not related to substrate specificity: ScaR presents three domains, while TroR and MntR present only the first two, yet the three of them recognize primarily Mn(II). IdeR and SirR present a FeoA-like C-terminal domain, but DtxR C-terminus does not, and the three proteins primarily bind iron. The metal binding sites for this family are likely to be not entirely conserved (Qiu et al., 1995; Schiering et al., 1995; Pohl et al., 1999; Feese et al., 2001; Wisedchaisri et al., 2004, 2007; Stoll et al., 2009). In DtxR, two metal binding sites were defined: the primary site, located at the interface between the DBD and the dimerization domain, and the ancillary site, formed between the dimerization domain and the C-terminal part of the protein. In IdeR, occupation of the first site causes dimerization, while binding to the second releases the repressor from its cognate operator (Chou et al., 2004). Mn binding TFs do not generally show equivalent binding sites. MntR contains two binding sites (A and C) located at the interface between the DBD and the dimerization domain (McGuire et al., 2013), while the molecular basis for metal specificity are unclear for TroR and ScaR. Structural data indicates that metal activation is different from DtxR (Hazlett et al., 2003; Stoll et al., 2009). Like in other metal sensory TFs, Ni(II) or Zn(II) binds MntR even with a greater affinity than its cognate metal Mn(II). This observation is in accordance with the Irving-William series for divalent metals, where the stability constant for complex formation follows the order Mg(II) < Mn(II) < Fe(II) < Co(II) < Ni(II) < Cu(II) > Zn(II) (Irving and Williams, 1948). However, *in vivo* MntR effector is indeed Mn(II), but not Ni(II) or Zn(II). The explanation to this apparent discrepancy is that Ni(II) or Zn(II) activate their specific eﬄux pumps before reaching the levels needed to repress Mn(II) uptake (Spiro and Dixon, 2010). This is something to take into account for the design of synthetic metal-specific WCBs.

A second family of TFs involved in the homeostasis of transition metals and also in the expression of virulence determinants is represented by the ferric uptake regulator Fur. Fur-like TFs are generally transcriptional repressors, although some transcriptional activators can be also found within the family (Delany et al., 2004; Troxell and Hassan, 2013). Other relevant members of Fur family include Mur, Nur, and Zur, which respond to manganese, nickel and zinc, respectively (Troxell and Hassan, 2013). Fur-like regulators are characterized by a simple structure (Fillat, 2014): a wHTH domain with a small C-terminal moiety that serves as metal-binding and dimerization interface (**Figure 8B**). Metal binding induces conformational changes that promote a tight association of Fur-like TFs with their operators, which are usually inverted repeats (Fillat, 2014). Besides the effector metal, most Fur-like TFs contain a second binding site for zinc, which serves a structural role in the protein (D’Autréaux et al., 2007).

A third family of TFs for transition metals comprises a set of homologous proteins for nickel homeostasis, generally known as NikR proteins (Schreiter et al., 2003; Chivers and Tahirov, 2005; West et al., 2010). NikR are tetramers organized in two domains: a tetramerization domain flanked by two dimeric RHH DNA-binding domains (**Figure 8C**). NikR contains four high-affinity sites within the tetramerization domain interface and secondary nickel-binding sites between the four subunits that form the tetramer. In *E. coli*, its proposed mechanism of action includes activation of the tetramer by Ni(II) occupation of the high affinity sites. Binding to secondary sites locks NikR in the closed conformation needed for interaction with DNA and repression of NikR regulated promoters (Chivers and Sauer, 2002; Chivers and Tahirov, 2005; Bahlawane et al., 2010). The operator for *E. coli* NikR is formed by a dyad-symmetric half-sites 5′-GTATGA-3′ on opposite ends of an imperfect 16–6–16 inverted repeat (Schreiter et al., 2003). When bound to the DNA, the RHH domains of the tetramer rotate around the flexible interdomain linkers to face the DNA binding motif toward the double strand. In this conformation, the antiparallel β-strands occupy the DNA major groove of an operator palindrome half-site (Schreiter et al., 2006). This regulatory mechanism is only partially conserved in other homologs. For example, *H. pylori* NikR conserves the high and the low affinity binding sites, but the later does not seem to have a regulatory role (Bahlawane et al., 2010; West et al., 2010). Also, despite sequence conservation, *H. pylori* NikR mechanism of DNA binding and regulation might not be identical to *E. coli*, since no consensus operator sequence has been identified and, in *H. pylori*, NikR displays a remarkable pleiotropic action (Bahlawane et al., 2010).

## Detection of Antibiotics

Antibiotics are among the most successful drugs used for human therapy. However, since they can challenge microbial populations, they must be considered as important pollutants as well (Martinez, 2009). Antibiotics are likely to be released into the aquatic environment via wastewater eﬄuent and agricultural runoff as a result of incomplete metabolism, ineffective treatment removal or improper disposal. Ultimately, large quantities of antibiotics are used annually in human therapy and in agriculture (Huang et al., 2011). The large excess of antibiotics released by human action into the environment has resulted in rampant levels of antibiotic resistance among bacterial populations, including many pathogens. This situation has triggered efforts to limit the usage of antibiotics in non-essential situations, such as animal husbandry (Andersson and Hughes, 2014). In this context, WCBs could be fundamental tools in containment efforts (Mungroo and Neethirajan, 2014). The TFs that respond to antibiotics are typically involved in the expression of antibiotic resistance genes or, less commonly, antibiotic production. Among these TFs, we distinguish between those that are antibiotic-specific, for which TetR represents the canonical prototype, and those involved in multiple antibiotic resistance, for which MarR constitutes the best characterized example.

## TetR Family

TetR family regulators (TFRs), named after the TF that regulates the operon involved in the resistance to tetracycline, represent one of the most common regulatory systems in bacteria. TFRs are mainly associated with antibiotic resistance and the regulation of genes encoding small-molecule exporters, although they also regulate other cellular functions (Ramos et al., 2005; Cuthbertson and Nodwell, 2013). TFRs have been found in almost every prokaryotic genera, usually with several members of the TFR per bacteria.

**FIGURE 9:**
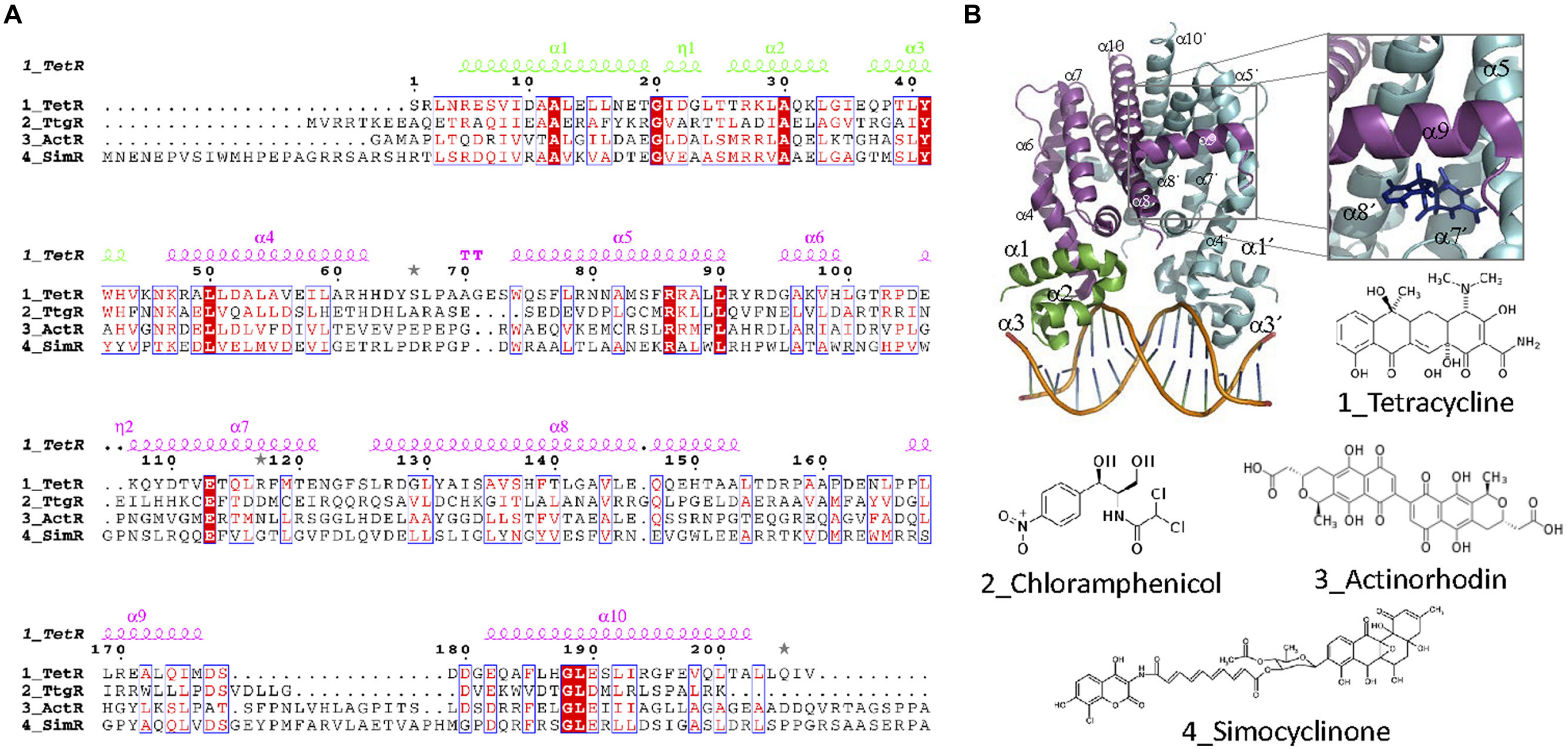
**TetR family. (A)** TetR family alignment. The alignment of representative TetR members for which effectors are antibiotics is shown. Secondary structure elements forming the DBD and the EDB are colored green and magenta, respectively. Alpha helices described in the text are labeled in the structure and in the alignment. **(B)** TFR structure. The crystal structure of the TFR TetR bound as a dimer to DNA (1QPI, Orth et al., 2000) is shown. The inset shows the location of a tetracycline derivative (iso-7-chlortetracycline) bound to TetR EDB (2X9D, Volkers et al., 2011). The molecular structure of antibiotic effectors recognized by the aligned proteins is also shown.

Structurally, TetR transcriptional regulators comprise an N-terminal DBD and a C-terminal EBD (**Figure 9**). Nine α helices are conserved in the structure of TFR. DBD, formed by helices 1–3, is highly conserved and contains a HTH motif. α3 is the recognition helix that is inserted in the major groove of the operator DNA. Several structures of TFRs bound to their cognate operators have been solved. The typical TFR operator contains a 15 bp IR with two 6 bp arms separated by 1 bp. The HTH motif of each monomer binds one of the IR arms. Like in other HTH IR DNA binding proteins, dimerization is required for activity. This structure was found in TetR (1QPI, Orth et al., 2000), SimR (2ZQL, Le et al., 2011), TM1030 (4I6Z), DesT (3LSR, Miller et al., 2010) and HrtR (3VOK, Sawai et al., 2012). In QacR, IR is unusually large for an operator sequence bound by a TFR, comprising 15 bp half-sites separated by a 6 bp spacer region (Grkovic et al., 2001). In fact, crystal structure of QacR bound to its 34 bp DNA operator (1JT0, Schumacher et al., 2002) is distinct from TetR and involves the binding of a pair of QacR dimers. Recognition at each IR half-site is mediated by a complement of DNA contacts made by two HTH motifs. Ms6564 (4JL3, Yang et al., 2013) and CgmR (2YVH, Itou et al., 2010) also binds DNA as tetramers. The EBD of TFRs, formed by helices 4–9, regulates DNA binding activity by interacting with its cognate inducer. In general, helices 5–7 form a central triangle, while helices 8 and 9 make up the dimerization interface, forming a four-helix bundle with the same helices from the other monomer (**Figures 9A,B**). Helices 4 and 6 of the ligand-binding domain link DBD and EDB domains. Effector binding to the EDB provokes a pendulum-like motion of helix 4 in a way that the HTH motifs are badly oriented for DNA binding. In TetR, (and also other homologs like TtgR and ActR) there is a “side entry” opening, distal to the dimerization interface, that seems to be the site of access for the effector (2TRT, Hinrichs et al., 1994); 2VKE, (Palm et al., 2007). Like in most TFs, the EDB is less conserved than the DBD (Ramos et al., 2005; Cuthbertson and Nodwell, 2013). In TFR, the variety of effectors is remarkably high. The structures of TFR bound to more than 100 ligands have been solved.

Among the spectrum of antibiotics recognized by TRFs, tetracycline and tetracycline-like antibiotics constitute the paradigmatic example, since the description of TetR in transposon Tn10 (Beck et al., 1982). Tetracyclines (**Figure 9**) are a class of broad-spectrum bacteriostatic antibiotics, as well as against intracellular organisms. They target the small subunit of the bacterial ribosome (Brodersen et al., 2000). TetA mediates tetracyclin detoxification by active eﬄux of the [MeTc]+ cation coupled to the uptake of a proton (Yamaguchi et al., 1991). TetR represses transcription of both its coding gene *tetR* and the resistance gene *tetA*. Other tetracycline-like antibiotics such as 7-chlorotetracycline (2TCT, Kisker et al., 1995), 7-iodotetracycline (2XB5, Hinrichs et al., 1994), 6-anhydrotetracycline (2VPR, Aleksandrov et al., 2008), minocycline (2XPV), oxytetracycline (2XPW), or 9-nitrotetracycline (4AUX) are also recognized by TetR.

Two other relevant TFRs, TgtR, and SimR, regulate the expression of antibiotic eﬄux pumps. Chloramphenicol (**Figure 9**) inhibits protein synthesis by binding reversibly to the 50S subunit of the bacterial ribosome. TtgR represses the transcription of TtgABC, a key eﬄux pump in *P. putida*, which causes resistance to antibiotics, solvents and toxic plant secondary products. TtgR repression is relieved by binding to Cm (2UXP, Alguel et al., 2007) along with other compounds like phloretin (2UXI), naringenin (2UXU), and quercetin (2UXH). Simocyclinones (**Figure 9**) are a new class of antibiotics that inhibit bacterial gyrase (Flatman et al., 2005). SimR represses the expression of SimX eﬄux pump. Repression is inhibited by binding of simocyclinone D8 to the EBD of SimR (2Y30, Le et al., 2010). In some cases, TFRs are involved in the control of the biosynthesis and export of antibiotics in producing strains, rather than regulating resistance operons. ActR, present in the biosynthetic gene cluster for the antibiotic actinorhodin (**Figure 9**) in *S. coelicolor*, controls the expression of two actinorhodin exporters (Tahlan et al., 2008). Both actinorhodin and its precursor can bind ActR and prevent its interaction with DNA (3B6A, Willems et al., 2008).

## MarR Family

**FIGURE 10:**
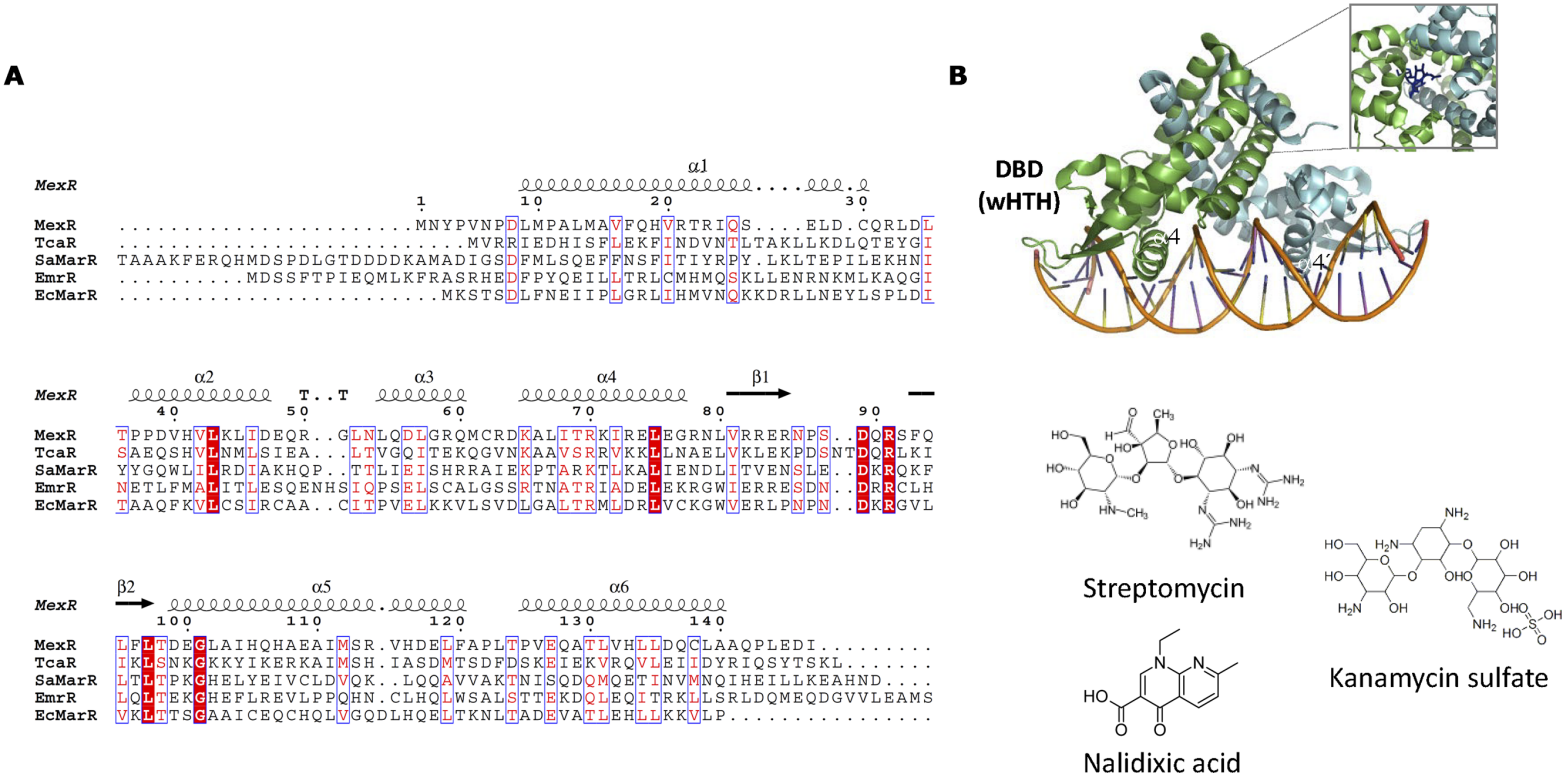
**MarR family. (A)** MarR family alignment. The alignment of representative MarR members is shown. Secondary structure elements described in the text are labeled in the structure and in the alignment. **(B)** MarR structure. The structure of *S. coelicolor* MarA bound to DNA (3ZPL, Chang et al., 2013; Stevenson et al., 2013) is shown as well as the location of kanamycin (4EM0) in the DNA-unbound repressor. The structure of MarR antibiotic effectors is represented.

The MarR (multiple antibiotic resistance regulator) family of prokaryotic transcriptional regulators (reviewed in Wilkinson and Grove, 2006) includes TFs involved in responses to antibiotic and oxidative stresses, and the catabolism of aromatic compounds. The prototype of the family, MarR, regulates the marRAB gene cluster, which confers resistance to multiple antibiotics (Alekshun and Levy, 1997). While in other TF families the DBD and EDB form structurally different domains, MarR is unique in that DBD and EBD almost completely overlap (**Figure 10**). The main component of the MarR family is the wHTH motif involved in DNA binding. MarR domain is disposed in the following order α1-α2-β1-α3-α4-β2-β3-α5-α6, being α4 the DNA recognition helix. In general, MarR family TFs are repressors that prevent RNApol recruitment by binding to operators that overlap with the –35 and/or –10 promoter elements. Although some MarR TFs bind directly to antibiotics (**Table 3**), these TFs tend to be promiscuous, responding to different anionic lipophilic molecules (Wilkinson and Grove, 2006). This relaxed specificity must be taken into account when MarR-like TFs are employed for biosensing purposes. For example, EmrR (MprA) negatively regulates the transcription of the multidrug resistance pump-encoding operon, emrRAB. Although nalidixic acid has been shown to be effector of EmrR, other organic compounds such as carbonyl cyanide, *m*-chlorophenyldrazone, 2,4-dinitrophenol or tetrachlorosalicylanilide have been shown to activate the emrRAB operon (Xiong et al., 2000). In some cases this effector promiscuity allows the TF to respond to a variety of different antibiotics. TcaR represses the *ica* locus, involved in poly-*N*-acetylglucosamine production and biofilm formation in *Staphylococcus epidermidis*. TcaR structure has been solved bound to salicylate (3KP6) or aminoglycosides and beta-lactam antibiotics such as methicillin (3KP4), kanamycin (3KP5), penicillin G (3KP2), ampicillin (3KP3), or streptomycin (3EJW; Chang et al., 2013).

**Table 3:** One-component TFs with antibiotic effectors.

Effector	Regulator	Regulated system	PDB	Reference
**TetRF**				
Tetracycline	TetR	TetA eﬄux pump	2TRT	Hinrichs et al. (1994)
Chloramphenicol	TtgR	TtgABC eﬄux pump	2UXP	Alguel et al. (2007)
Actinorhodin	ActR	ActA eﬄux pump	3B6A	Willems et al. (2008)
Ethionamine boosters	EthR	EthA flavoprotein monooxygenase	1T56,	Dover et al. (2004)
Simocyclinone	SimR	SimX eﬄux pump	2Y30	Le et al. (2010)
**MarRF**				
Salicylate, Tetracycline, Chloramphenicol	MarR	MarA activation of AcrAB-TolC multidrug eﬄux system	1JGS	Alekshun et al. (2001)
Peroxide stress	MexR	Multidrug eﬄux pump	1LNW	Lim et al. (2002)
Kanamycin	SAR2349	Uncharacterized	4EM0	Chang et al. (2013)
Streptomycin	TcaR	GlcNAc production, biofilm formation	4EJW	Chang et al. (2013)
Nalidixic acid	EmrR	Multidrug resistance pump		Xiong et al. (2000)

## Future Perspectives

In this review, we focused on aromatic compounds, metals, and antibiotics because these are common sources of environmental contamination, so they have been amply tested for bacterial sensor devices. Since there is an almost inexhaustible repertoire of different molecules recognized by bacterial TFs, which will provide us with additional sources of synthetic devices, we hope the examples given here will be useful for future mining and characterization. Nature will always provide new mechanisms of action and systems with new properties if mined with an open eye.

The wide number of analytes recognized by TFs is good news for synthetic biology, in that it expands the repertoire of potential input signals that can be used for sensor device construction. We hope our work also clarified that each TF family operates in an idiosyncratic way, so that overall generalizations are difficult and simplifications are likely to produce wrong results. Overall, we identify four main challenges ahead for the systematic employment of TFs in WCBs. The first one involves the quantitative characterization of signal transduction. Robust WCBs demand each TF/analyte pair should be characterized by its transfer function (Fernandez-Lopez et al., 2010) under a number of relevant conditions. The second challenge is to rationally engineer or evolve TFs with enhanced sensitivity and specificity. As we have seen, evolutionary exaptation not always results in highly specific TFs. Most commonly, sensory TFs have a primary effector but also respond to other molecules. In other cases TFs respond to intermediate metabolites of environmental pollutants rather than the pollutant itself. Rational engineering requires further knowledge of the biochemical basis of substrate specificity. Alternatively, directed evolution strategies could be used to select for TFs with the desired specificity profiles. A third challenge involves the development of complex circuits able to perform logic operations. This demands TF/promoter pairs that can be used in a combinatorial fashion. For this purpose, comparative scales should be produced, so the dynamic range of each TF/signal pair can be assessed with respect to each other. This will allow complex circuit building, ensuring that the dynamic range of the upstream output device is within range of the input of the downstream device. In this way, impedance matching problems will be avoided and fine tuning of connected devices will become unnecessary (Yokobayashi et al., 2002; Moon et al., 2012). Finally, the practical applications of WCBs have been hampered by biosafety concerns. WCBs are genetically modified organisms, which require bio-containment strategies to prevent their proliferation in the environment. Thus, regulatory issues will need to be clarified before WCBs can achieve their full potential as highly sensitive, inexpensive sensors for field use (French et al., 2011).

Whole cell biosensors development will critically depend on our ability to develop reliable and secure bacterial chassis, i.e., the generation of bacterial strains with engineered firewalls for biocontainment and prevention of horizontal gene transfer. In this respect, the generation of an engineered *E. coli* strain that requires non-standard amino acids for survival (Mandell et al., 2015) constitutes a promising development. Resistant to evolutionary escape through mutagenesis and horizontal gene transfer, this strain could be an excellent chassis for biosensor systems. In summary, engineered WCBs produced by synthetic biology constitute one of the pillars for bacterial domestication and thus for the inauguration of a new era in human civilization (Church and Regis, 2012). Ours is a humble attempt to enlighten progress in this direction.

## Conflict of Interest Statement

The authors declare that the research was conducted in the absence of any commercial or financial relationships that could be construed as a potential conflict of interest.

## References

[B1] Aleksandrov A. Schuldt L. Hinrichs W. Simonson T. (2008). Tet repressor induction by tetracycline: a molecular dynamics, continuum electrostatics, and crystallographic study. *J. Mol. Biol.* 378 898–912. 10.1016/j.jmb.2008.03.022 18395746

[B2] Alekshun M. N. Levy S. B. (1997). Regulation of chromosomally mediated multiple antibiotic resistance: the mar regulon. *Antimicrob. Agents Chemother.* 41 2067–2075. 9333027 10.1128/aac.41.10.2067 PMC164072

[B3] Alekshun M. N. Levy S. B. Mealy T. R. Seaton B. A. Head J. F. (2001). The crystal structure of MarR, a regulator of multiple antibiotic resistance, at 2.3 A resolution. *Nat. Struct. Biol.* 8 710–714. 10.1038/90429 11473263

[B4] Alguel Y. Meng C. Terán W. Krell T. Ramos J. L. Gallegos M.-T. (2007). Crystal structures of multidrug binding protein TtgR in complex with antibiotics and plant antimicrobials. *J. Mol. Biol.* 369 829–840. 10.1016/j.jmb.2007.03.062 17466326 PMC2756574

[B5] Amábile-Cuevas C. F. Demple B. (1991). Molecular characterization of the soxRS genes of *Escherichia coli*: two genes control a superoxide stress regulon. *Nucleic Acids Res.* 19 4479–4484. 10.1093/nar/19.16.4479 1653416 PMC328637

[B6] Amaro F. Turkewitz A. P. Martín-González A. Gutiérrez J. C. (2014). Functional GFP-metallothionein fusion protein from *Tetrahymena thermophila*: a potential whole-cell biosensor for monitoring heavy metal pollution and a cell model to study metallothionein overproduction effects. *Biometals* 27 195–205. 10.1007/s10534-014-9704-0 24430977 PMC4707044

[B7] An Y. J. Ahn B.-E. Han A.-R. Kim H.-M. Chung K. M. Shin J.-H. (2009). Structural basis for the specialization of Nur, a nickel-specific Fur homolog, in metal sensing and DNA recognition. *Nucleic Acids Res.* 37 3442–3451. 10.1093/nar/gkp198 19336416 PMC2691836

[B8] Andersson D. I. Hughes D. (2014). Microbiological effects of sublethal levels of antibiotics. *Nat. Rev. Microbiol.* 12 465–478. 10.1038/nrmicro3270 24861036

[B9] Andrews S. C. Robinson A. K. Rodríguez-Quiñones F. (2003). Bacterial iron homeostasis. *FEMS Microbiol. Rev.* 27 215–237. 10.1016/S0168-6445(03)00055-X 12829269

[B10] Ansari A. Z. Bradner J. E. O’Halloran T. V. (1995). DNA-bend modulation in a repressor-to-activator switching mechanism. *Nature* 374 371–375. 10.1038/374370a0 7885478

[B11] Bahlawane C. Dian C. Muller C. Round A. Fauquant C. Schauer K. (2010). Structural and mechanistic insights into *Helicobacter pylori* NikR activation. *Nucleic Acids Res.* 38 3106–3118. 10.1093/nar/gkp1216 20089510 PMC2875016

[B12] Barrineau P. Gilbert P. Jackson W. J. Jones C. S. Summers A. O. Wisdom S. (1985). “The structure of the mer operon,” in *Plasmids in Bacteria* eds Helinski D. R. Cohen S. N. Clewell D. B. Jackson D. A. Hollaender A. (New York, NY: Springer) 707–718. 10.1007/978-1-4613-2447-8_49 2990435

[B13] Beck C. F. Mutzel R. Barbé J. Müller W. (1982). A multifunctional gene (tetR) controls Tn10-encoded tetracycline resistance. *J. Bacteriol.* 150 633–642. 6279565 10.1128/jb.150.2.633-642.1982 PMC216410

[B14] Bell K. S. Philp J. C. Aw D. W. J. Christofi N. (1998). A review: the genus *Rhodococcus*. *J. Appl. Microbiol.* 85 195–210. 10.1046/j.1365-2672.1998.00525.x 9750292

[B15] Bontidean I. Mortari A. Leth S. Brown N. L. Karlson U. Larsen M. M. (2004). Biosensors for detection of mercury in contaminated soils. *Environ. Pollut.* 131 255–262. 10.1016/j.envpol.2004.02.019 15234092

[B16] Brennan R. G. Matthews B. W. (1989). The helix-turn-helix DNA binding motif. *J. Biol. Chem.* 264 1903–1906. 2644244

[B17] Brocklehurst K. R. Hobman J. L. Lawley B. Blank L. Marshall S. J. Brown N. L. (1999). ZntR is a Zn(II)-responsive MerR-like transcriptional regulator of zntA in *Escherichia coli*. *Mol. Microbiol.* 31 893–902. 10.1046/j.1365-2958.1999.01229.x 10048032

[B18] Brodersen D. E. Clemons W. M. Carter A. P. Morgan-Warren R. J. Wimberly B. T. Ramakrishnan V. (2000). The structural basis for the action of the antibiotics tetracycline, pactamycin, and hygromycin B on the 30S ribosomal subunit. *Cell* 103 1143–1154. 10.1016/S0092-8674(00)00216-6 11163189

[B19] Brown N. L. Stoyanov J. V. Kidd S. P. Hobman J. L. (2003). The MerR family of transcriptional regulators. *FEMS Microbiol. Rev.* 27 145–163. 10.1016/S0168-6445(03)00051-2 12829265

[B20] Bruins M. R. Kapil S. Oehme F. W. (2000). Microbial resistance to metals in the environment. *Ecotoxicol. Environ. Saf.* 45 198–207. 10.1006/eesa.1999.1860 10702338

[B21] Bundy B. M. Collier L. S. Hoover T. R. Neidle E. L. (2002). Synergistic transcriptional activation by one regulatory protein in response to two metabolites. *Proc. Natl. Acad. Sci. U.S.A.* 99 7693–7698. 10.1073/pnas.102605799 12032345 PMC124324

[B22] Busenlehner L. S. Pennella M. A. Giedroc D. P. (2003). The SmtB/ArsR family of metalloregulatory transcriptional repressors: structural insights into prokaryotic metal resistance. *FEMS Microbiol. Rev.* 27 131–143. 10.1016/S0168-6445(03)00054-8 12829264

[B23] Bush M. Dixon R. (2012). The role of bacterial enhancer binding proteins as specialized activators of σ54-dependent transcription. *Microbiol. Mol. Biol. Rev.* 76 497–529. 10.1128/MMBR.00006-12 22933558 PMC3429621

[B24] Bustos S. A. Schleif R. F. (1993). Functional domains of the AraC protein. *Proc. Natl. Acad. Sci. U. S. A.* 90 5638–5642. 10.1073/pnas.90.12.5638 8516313 PMC46776

[B25] Calles B. de Lorenzo V. (2013). Expanding the boolean logic of the prokaryotic transcription factor XylR by functionalization of permissive sites with a protease-target sequence. *ACS Synth. Biol.* 2 594–603. 10.1021/sb400050k 23875967

[B26] Campos V. L. Zaror C. A. Mondaca M. A. (2004). Detection of chlorinated phenols in kraft pulp bleaching eﬄuents using DmpR mutant strains. *Bull. Environ. Contam. Toxicol.* 73 666–673. 10.1007/s00128-004-0478-x 15389331

[B27] Cerminati S. Soncini F. C. Checa S. K. (2011). Selective detection of gold using genetically engineered bacterial reporters. *Biotechnol. Bioeng.* 108 2553–2560. 10.1002/bit.23213 21618467

[B28] Chalfie M. Tu Y. Euskirchen G. Ward W. W. Prasher D. C. (1994). Green fluorescent protein as a marker for gene expression. *Science* 263 802–805. 10.1126/science.8303295 8303295

[B29] Chang Y.-M. Chen C. K.-M. Ko T.-P. Chang-Chien M. W. Wang A. H.-J. (2013). Structural analysis of the antibiotic-recognition mechanism of MarR proteins. *Acta Crystallogr. Sect. D Biol. Crystallogr.* 69 1138–1149. 10.1107/S0907444913007117 23695258

[B30] Changela A. Chen K. Xue Y. Holschen J. Outten C. E. O’Halloran T. V. (2003). Molecular basis of metal-ion selectivity and zeptomolar sensitivity by CueR. *Science* 301 1383–1387. 10.1126/science.1085950 12958362

[B31] Cherney L. T. Cherney M. M. Garen C. R. James M. N. G. (2010). Crystal structure of the intermediate complex of the arginine repressor from *Mycobacterium tuberculosis* bound with its DNA operator reveals detailed mechanism of arginine repression. *J. Mol. Biol.* 399 240–254. 10.1016/j.jmb.2010.03.065 20382162

[B32] Chivers P. T. Sauer R. T. (2002). NikR repressor: high-affinity nickel binding to the C-terminal domain regulates binding to operator DNA. *Chem. Biol.* 9 1141–1148. 10.1016/S1074-5521(02)00241-7 12401498

[B33] Chivers P. T. Tahirov T. H. (2005). Structure of *Pyrococcus horikoshii* NikR: nickel sensing and implications for the regulation of DNA recognition. *J. Mol. Biol.* 348 597–607. 10.1016/j.jmb.2005.03.017 15826657

[B34] Chou C. J. Wisedchaisri G. Monfeli R. R. Oram D. M. Holmes R. K. Hol W. G. J. (2004). Functional studies of the *Mycobacterium tuberculosis* iron-dependent regulator. *J. Biol. Chem.* 279 53554–53561. 10.1074/jbc.M407385200 15456786

[B35] Church G. M. Regis E. (2012). *Regenesis: How Synthetic Biology Will Reinvent Nature and Ourselves.* New York, NY: Basic Books.

[B36] Clark T. J. Momany C. Neidle E. L. (2002). The benPK operon, proposed to play a role in transport, is part of a regulon for benzoate catabolism in *Acinetobacter* sp. strain ADP1. *Microbiol. Read. Engl.* 148 1213–1223. 10.1099/00221287-148-4-1213 11932465

[B37] Cook W. J. Kar S. R. Taylor K. B. Hall L. M. (1998). Crystal structure of the cyanobacterial metallothionein repressor SmtB: a model for metalloregulatory proteins. *J. Mol. Biol.* 275 337–346. 10.1006/jmbi.1997.1443 9466913

[B38] Craven S. H. Ezezika O. C. Haddad S. Hall R. A. Momany C. Neidle E. L. (2009). Inducer responses of BenM, a LysR-type transcriptional regulator from *Acinetobacter baylyi* ADP1. *Mol. Microbiol.* 72 881–894. 10.1111/j.1365-2958.2009.06686.x 19400783

[B39] Cuthbertson L. Nodwell J. R. (2013). The TetR family of regulators. *Microbiol. Mol. Biol. Rev.* 77 440–475. 10.1128/MMBR.00018-13 24006471 PMC3811609

[B40] D’Autréaux B. Pecqueur L. Gonzalez de Peredo A. Diederix R. E. M. Caux-Thang C. Tabet L. (2007). Reversible redox- and zinc-dependent dimerization of the *Escherichia coli* fur protein. *Biochemistry* 46 1329–1342. 10.1021/bi061636r 17260962

[B41] De Carlo S. Chen B. Hoover T. R. Kondrashkina E. Nogales E. Nixon B. T. (2006). The structural basis for regulated assembly and function of the transcriptional activator NtrC. *Genes Dev.* 20 1485–1495. 10.1101/gad.1418306 16751184 PMC1475761

[B42] Delany I. Rappuoli R. Scarlato V. (2004). Fur functions as an activator and as a repressor of putative virulence genes in *Neisseria meningitidis*. *Mol. Microbiol.* 52 1081–1090. 10.1111/j.1365-2958.2004.04030.x 15130126

[B43] de Las Heras A. Chavarría M. de Lorenzo V. (2011). Association of dnt genes of *Burkholderia* sp. *DNT* with the substrate-blind regulator DntR draws the evolutionary itinerary of 2,4-dinitrotoluene biodegradation. *Mol. Microbiol.* 82 287–299. 10.1111/j.1365-2958.2011.07825.x 21923773

[B44] de Las Heras A. de Lorenzo V. (2011a). Cooperative amino acid changes shift the response of the σ54-dependent regulator XylR from natural m-xylene towards xenobiotic 2,4-dinitrotoluene. *Mol. Microbiol.* 79 1248–1259. 10.1111/j.1365-2958.2010.07518.x 21205010

[B45] de las Heras A. de Lorenzo V. (2011b). In situ detection of aromatic compounds with biosensor *Pseudomonas putida* cells preserved and delivered to soil in water-soluble gelatin capsules. *Anal. Bioanal. Chem.* 400 1093–1104. 10.1007/s00216-010-4558-y 21174197

[B46] de Las Heras A. Fraile S. de Lorenzo V. (2012). Increasing signal specificity of the TOL network of *Pseudomonas putida* mt-2 by rewiring the connectivity of the master regulator XylR. *PLoS Genet* 8:e1002963 10.1371/journal.pgen.1002963 PMC3469447 23071444

[B47] Devesse L. Smirnova I. Lönneborg R. Kapp U. Brzezinski P. Leonard G. A. (2011). Crystal structures of DntR inducer binding domains in complex with salicylate offer insights into the activation of LysR-type transcriptional regulators. *Mol. Microbiol.* 81 354–367. 10.1111/j.1365-2958.2011.07673.x 21692874

[B48] Devos D. Garmendia J. de Lorenzo V. Valencia A. (2002). Deciphering the action of aromatic effectors on the prokaryotic enhancer-binding protein XylR: a structural model of its N-terminal domain. *Environ. Microbiol.* 4 29–41. 10.1046/j.1462-2920.2002.00265.x 11966823

[B49] Ding H. Hidalgo E. Demple B. (1996). The redox state of the [2Fe-2S] clusters in SoxR protein regulates its activity as a transcription factor. *J. Biol. Chem.* 271 33173–33175. 10.1074/jbc.271.52.33173 8969171

[B50] Domínguez-Cuevas P. Marín P. Busby S. Ramos J. L. Marqués S. (2008). Roles of effectors in XylS-dependent transcription activation: intramolecular domain derepression and DNA binding. *J. Bacteriol.* 190 3118–3128. 10.1128/JB.01784-07 18296514 PMC2347401

[B51] Dover L. G. Corsino P. E. Daniels I. R. Cocklin S. L. Tatituri V. Besra G. S. (2004). Crystal structure of the TetR/CamR family repressor *Mycobacterium tuberculosis* EthR implicated in ethionamide resistance. *J. Mol. Biol.* 340 1095–1105. 10.1016/j.jmb.2004.06.003 15236969

[B52] Eicken C. Pennella M. A. Chen X. Koshlap K. M. VanZile M. L. Sacchettini J. C. (2003). A metal-ligand-mediated intersubunit allosteric switch in related SmtB/ArsR zinc sensor proteins. *J. Mol. Biol.* 333 683–695. 10.1016/j.jmb.2003.09.007 14568530

[B53] Ezezika O. C. Haddad S. Clark T. J. Neidle E. L. Momany C. (2007). Distinct effector-binding sites enable synergistic transcriptional activation by BenM, a LysR-type regulator. *J. Mol. Biol.* 367 616–629. 10.1016/j.jmb.2006.09.090 17291527

[B54] Feese M. D. Ingason B. P. Goranson-Siekierke J. Holmes R. K. Hol W. G. (2001). Crystal structure of the iron-dependent regulator from *Mycobacterium tuberculosis* at 2.0-A resolution reveals the Src homology domain 3-like fold and metal binding function of the third domain. *J. Biol. Chem.* 276 5959–5966. 10.1074/jbc.M007531200 11053439

[B55] Fernandez-Lopez R. Del Campo I. Ruiz R. Lanza V. Vielva L. de la Cruz F. (2010). Numbers on the edges: a simplified and scalable method for quantifying the gene regulation function. *BioEssays* 32 346–355. 10.1002/bies.200900164 20349442

[B56] Fillat M. F. (2014). The FUR (ferric uptake regulator) superfamily: diversity and versatility of key transcriptional regulators. *Arch. Biochem. Biophys.* 546 41–52. 10.1016/j.abb.2014.01.029 24513162

[B57] Flatman R. H. Howells A. J. Heide L. Fiedler H.-P. Maxwell A. (2005). Simocyclinone D8, an Inhibitor of DNA Gyrase with a novel mode of action. *Antimicrob. Agents Chemother.* 49 1093–1100. 10.1128/AAC.49.3.1093-1100.2005 15728908 PMC549283

[B58] Fowler A. V. Zabin I. (1978). Amino acid sequence of beta-galactosidase. XI. Peptide ordering procedures and the complete sequence. *J. Biol. Chem.* 253 5521–5525. 97298

[B59] French C. E. de Mora K. Joshi N. Elfick A. Haseloff J. Ajioka J. (2011). *Synthetic Biology And The Art Of Biosensor Design.* Available at: http://www.ncbi.nlm.nih.gov/books/NBK84465/ [accessed January 21, 2015].

[B60] Gajiwala K. S. Burley S. K. (2000). Winged helix proteins. *Curr. Opin. Struct. Biol.* 10 110–116. 10.1016/S0959-440X(99)00057-3 10679470

[B61] Gallegos M. T. Schleif R. Bairoch A. Hofmann K. Ramos J. L. (1997). Arac/XylS family of transcriptional regulators. *Microbiol. Mol. Biol. Rev.* 61 393–410. 9409145 10.1128/mmbr.61.4.393-410.1997 PMC232617

[B62] Galvão T. C. de Lorenzo V. (2006). Transcriptional regulators à la carte: engineering new effector specificities in bacterial regulatory proteins. *Curr. Opin. Biotechnol.* 17 34–42. 10.1016/j.copbio.2005.12.002 16359854

[B63] Garmendia J. de las Heras A. Galvão T. C. de Lorenzo V. (2008). Tracing explosives in soil with transcriptional regulators of *Pseudomonas putida* evolved for responding to nitrotoluenes. *Microb. Biotechnol.* 1 236–246. 10.1111/j.1751-7915.2008.00027.x 21261843 PMC3815885

[B64] Ghim C.-M. Lee S. K. Takayama S. Mitchell R. J. (2010). The art of reporter proteins in science: past, present and future applications. *BMB Rep.* 43 451–460. 10.5483/BMBRep.2010.43.7.451 20663405

[B65] Gilston B. A. Wang S. Marcus M. D. Canalizo-Hernández M. A. Swindell E. P. Xue Y. (2014). Structural and mechanistic basis of zinc regulation across the *E. coli* Zur regulon. *PLoS Biol.* 12:e1001987 10.1371/journal.pbio.1001987 PMC4219657 25369000

[B66] Grkovic S. Brown M. H. Schumacher M. A. Brennan R. G. Skurray R. A. (2001). The *Staphylococcal* QacR multidrug regulator binds a correctly spaced operator as a pair of dimers. *J. Bacteriol.* 183 7102–7109. 10.1128/JB.183.24.7102-7109.2001 11717268 PMC95558

[B67] Guo H.-B. Johs A. Parks J. M. Olliff L. Miller S. M. Summers A. O. (2010). Structure and conformational dynamics of the metalloregulator MerR upon binding of Hg(II). *J. Mol. Biol.* 398 555–568. 10.1016/j.jmb.2010.03.020 20303978

[B68] Gupta S. Saxena M. Saini N. Mahmooduzzafar Kumar R. Kumar A. (2012). An effective strategy for a whole-cell biosensor based on putative effector interaction site of the regulatory DmpR protein. *PLoS ONE* 7:e43527 10.1371/journal.pone.0043527 PMC3427379 22937060

[B69] Gutiérrez J. C. Amaro F. Martín-González A. (2015). Heavy metal whole-cell biosensors using eukaryotic microorganisms: an updated critical review. *Front. Microbiol.* 6:48 10.3389/fmicb.2015.00048 PMC4335268 25750637

[B70] Hakkila K. Maksimow M. Karp M. Virta M. (2002). Reporter genes *lucFF, luxCDABE, gfp*, and *dsred* have different characteristics in whole-cell bacterial sensors. *Anal. Biochem.* 301 235–242. 10.1006/abio.2001.5517 11814294

[B71] Hayes R. P. Moural T. W. Lewis K. M. Onofrei D. Xun L. Kang C. (2014). Structures of the inducer-binding domain of pentachlorophenol-degrading gene regulator PcpR from *Sphingobium chlorophenolicum*. *Int. J. Mol. Sci.* 15 20736–20752. 10.3390/ijms151120736 25397598 PMC4264193

[B72] Hazlett K. R. O. Rusnak F. Kehres D. G. Bearden S. W. La Vake C. J. La Vake M. E. (2003). The *Treponema pallidum* tro operon encodes a multiple metal transporter, a zinc-dependent transcriptional repressor, and a semi-autonomously expressed phosphoglycerate mutase. *J. Biol. Chem.* 278 20687–20694. 10.1074/jbc.M300781200 12668673

[B73] Hill P. J. Cockayne A. Landers P. Morrissey J. A. Sims C. M. Williams P. (1998). SirR, a novel iron-dependent repressor in *Staphylococcus epidermidis*. *Infect. Immun.* 66 4123–4129. 9712757 10.1128/iai.66.9.4123-4129.1998 PMC108495

[B74] Hinrichs W. Kisker C. Düvel M. Müller A. Tovar K. Hillen W. (1994). Structure of the tet repressor-tetracycline complex and regulation of antibiotic resistance. *Science* 264 418–420. 10.1126/science.8153629 8153629

[B75] Hirschman J. Wong P. K. Sei K. Keener J. Kustu S. (1985). Products of nitrogen regulatory genes ntrA and ntrC of enteric bacteria activate glnA transcription in vitro: evidence that the ntrA product is a sigma factor. *Proc. Natl. Acad. Sci. U.S.A.* 82 7525–7529. 10.1073/pnas.82.22.7525 2999766 PMC390849

[B76] Holmes D. J. Caso J. L. Thompson C. J. (1993). Autogenous transcriptional activation of a thiostrepton-induced gene in Streptomyces lividans. *EMBO J.* 12 3183–3191. 7688297 10.1002/j.1460-2075.1993.tb05987.x PMC413584

[B77] Huang C.-H. Renew J. E. Smeby K. L. Pinkston K. Sedlak D. L. (2011). Assessment of potential antibiotic contaminants in water and preliminary occurrence analysis. *J. Contemp. Water Res. Educ.* 120 30–40.

[B78] Hunt T. P. Magasanik B. (1985). Transcription of glnA by purified *Escherichia coli* components: core RNA polymerase and the products of glnF, glnG, and glnL. *Proc. Natl. Acad. Sci. U.S.A.* 82 8453–8457. 10.1073/pnas.82.24.8453 2867543 PMC390934

[B79] Inouye S. Nakazawa A. Nakazawa T. (1981). Molecular cloning of gene xylS of the TOL plasmid: evidence for positive regulation of the xylDEGF operon by xylS. *J. Bacteriol.* 148 413–418. 6271729 10.1128/jb.148.2.413-418.1981 PMC216221

[B80] Irving H. Williams R. (1948). Order of stability of metal complexes. *Nature* 162 746–747. 10.1038/162746a0

[B81] Itou H. Watanabe N. Yao M. Shirakihara Y. Tanaka I. (2010). Crystal structures of the multidrug binding repressor *Corynebacterium glutamicum* CgmR in complex with inducers and with an operator. *J. Mol. Biol.* 403 174–184. 10.1016/j.jmb.2010.07.042 20691702

[B82] Itou H. Yao M. Watanabe N. Tanaka I. (2008). Crystal structure of the PH1932 protein, a unique archaeal ArsR type winged-HTH transcription factor from *Pyrococcus horikoshii* OT3. *Proteins* 70 1631–1634. 10.1002/prot.21851 18076033

[B83] Kalnins A. Otto K. Rüther U. Müller-Hill B. (1983). Sequence of the lacZ gene of *Escherichia coli*. *EMBO J.* 2 593–597. 6313347 10.1002/j.1460-2075.1983.tb01468.x PMC555066

[B84] Kehl-Fie T. E. Skaar E. P. (2010). Nutritional immunity beyond iron: a role for manganese and zinc. *Curr. Opin. Chem. Biol.* 14 218–224. 10.1016/j.cbpa.2009.11.008 20015678 PMC2847644

[B85] Kelley L. A. Sternberg M. J. E. (2009). Protein structure prediction on the Web: a case study using the Phyre server. *Nat. Protoc.* 4 363–371. 10.1038/nprot.2009.2 19247286

[B86] Kisker C. Hinrichs W. Tovar K. Hillen W. Saenger W. (1995). The complex formed between Tet repressor and tetracycline-Mg2+ reveals mechanism of antibiotic resistance. *J. Mol. Biol.* 247 260–280. 10.1006/jmbi.1994.0138 7707374

[B87] Kustu S. Santero E. Keener J. Popham D. Weiss D. (1989). Expression of sigma 54 (ntrA)-dependent genes is probably united by a common mechanism. *Microbiol. Rev.* 53 367–376. 2677638 10.1128/mr.53.3.367-376.1989 PMC372741

[B88] Landrain T. E. Carrera J. Kirov B. Rodrigo G. Jaramillo A. (2009). Modular model-based design for heterologous bioproduction in bacteria. *Curr. Opin. Biotechnol.* 20 272–279. 10.1016/j.copbio.2009.06.003 19559595

[B89] Laub M. T. Goulian M. (2007). Specificity in two-component signal transduction pathways. *Annu. Rev. Genet.* 41 121–145. 10.1146/annurev.genet.41.042007.170548 18076326

[B90] Le T. B. K. Schumacher M. A. Lawson D. M. Brennan R. G. Buttner M. J. (2011). The crystal structure of the TetR family transcriptional repressor SimR bound to DNA and the role of a flexible N-terminal extension in minor groove binding. *Nucleic Acids Res.* 39 9433–9447. 10.1093/nar/gkr640 21835774 PMC3241653

[B91] Le T. B. K. Stevenson C. E. M. Fiedler H.-P. Maxwell A. Lawson D. M. Buttner M. J. (2010). Structures of the Tetr-Like simocyclinone eﬄux pump repressor, Simr, and the mechanism of Ligand-mediated derepression. *J. Mol. Biol.* 408 40–56. 10.1016/j.jmb.2011.02.035 21354180

[B92] Lee C. W. Chakravorty D. K. Chang F.-M. J. Reyes-Caballero H. Ye Y. Merz K. M. (2012). Solution structure of *Mycobacterium tuberculosis* NmtR in the apo state: insights into Ni(II)-mediated allostery. *Biochemistry* 51 2619–2629. 10.1021/bi3001402 22394357 PMC3500661

[B93] Lee S.-Y. De La Torre A. Yan D. Kustu S. Nixon B. T. Wemmer D. E. (2003). Regulation of the transcriptional activator NtrC1: structural studies of the regulatory and AAA+ ATPase domains. *Genes Dev.* 17 2552–2563. 10.1101/gad.1125603 14561776 PMC218149

[B94] Lim D. Poole K. Strynadka N. C. J. (2002). Crystal structure of the MexR repressor of themexRAB-oprM multidrug eﬄux operon of *Pseudomonas aeruginosa*. *J. Biol. Chem.* 277 29253–29259. 10.1074/jbc.M111381200 12034710

[B95] Liu Y. Li W. Wei Y. Jiang Y. Tan X. (2013). Efficient preparation and metal specificity of the regulatory protein TroR from the human pathogen *Treponema pallidum*. *Metallomics* 5 1448–1457. 10.1039/c3mt00163f 23945957

[B96] Lönneborg R. Varga E. Brzezinski P. (2012). Directed evolution of the transcriptional regulator DntR: isolation of mutants with improved DNT-response. *PLoS ONE* 7:e29994 10.1371/journal.pone.0029994 PMC3261848 22276138

[B97] Lowden M. J. Skorupski K. Pellegrini M. Chiorazzo M. G. Taylor R. K. Kull F. J. (2010). Structure of *Vibrio cholerae* ToxT reveals a mechanism for fatty acid regulation of virulence genes. *Proc. Natl. Acad. Sci. U.S.A.* 107 2860–2865. 10.1073/pnas.0915021107 20133655 PMC2840316

[B98] Macia J. Sole R. (2014). How to make a synthetic multicellular computer. *PLoS ONE* 9:e81248 10.1371/journal.pone.0081248 PMC3929274 24586222

[B99] Maddocks S. E. Oyston P. C. F. (2008). Structure and function of the LysR-type transcriptional regulator (LTTR) family proteins. *Microbiol. Read. Engl.* 154 3609–3623. 10.1099/mic.0.2008/022772-0 19047729

[B100] Mandell D. J. Lajoie M. J. Mee M. T. Takeuchi R. Kuznetsov G. Norville J. E. (2015). Biocontainment of genetically modified organisms by synthetic protein design. *Nature* 518 55–60. 10.1038/nature14121 25607366 PMC4422498

[B101] Martinez J. L. (2009). Environmental pollution by antibiotics and by antibiotic resistance determinants. *Environ. Pollut.* 157 2893–2902. 10.1016/j.envpol.2009.05.051 19560847

[B102] McGuire A. M. Cuthbert B. J. Ma Z. Grauer-Gray K. D. Brunjes Brophy M. Spear K. A. (2013). Roles of the A and C sites in the manganese-specific activation of MntR. *Biochemistry* 52 701–713. 10.1021/bi301550t 23298157 PMC3562352

[B103] Michan C. Zhou L. Gallegos M. T. Timmis K. N. Ramos J. L. (1992). Identification of critical amino-terminal regions of XylS. The positive regulator encoded by the TOL plasmid. *J. Biol. Chem.* 267 22897–22901. 1429638

[B104] Michelini E. Cevenini L. Calabretta M. M. Spinozzi S. Camborata C. Roda A. (2013). Field-deployable whole-cell bioluminescent biosensors: so near and yet so far. *Anal. Bioanal. Chem.* 405 6155–6163. 10.1007/s00216-013-7043-6 23739749

[B105] Miller D. J. Zhang Y.-M. Subramanian C. Rock C. O. White S. W. (2010). Structural basis for the transcriptional regulation of membrane lipid homeostasis. *Nat. Struct. Mol. Biol.* 17 971–975. 10.1038/nsmb.1847 20639888 PMC2935088

[B106] Mi Na Kim H. H. P. (2005). Construction and comparison of *Escherichia coli* whole-cell biosensors capable of detecting aromatic compounds. *J. Microbiol. Methods* 60 235–45. 10.1016/j.mimet.2004.09.018 15590098

[B107] Monferrer D. Tralau T. Kertesz M. A. Dix I. Solà M. Usón I. (2010). Structural studies on the full-length LysR-type regulator TsaR from *Comamonas testosteroni* T-2 reveal a novel open conformation of the tetrameric LTTR fold. *Mol. Microbiol.* 75 1199–1214. 10.1111/j.1365-2958.2010.07043.x 20059681

[B108] Moon T. S. Lou C. Tamsir A. Stanton B. C. Voigt C. A. (2012). Genetic programs constructed from layered logic gates in single cells. *Nature* 491 249–253. 10.1038/nature11516 23041931 PMC3904217

[B109] Mukherjee D. Datta A. B. Chakrabarti P. (2014). Crystal structure of HlyU, the hemolysin gene transcription activator, from *Vibrio cholerae* N16961 and functional implications. *Biochim. Biophys. Acta* 1844 2346–2354. 10.1016/j.bbapap.2014.09.020 25450504

[B110] Mungroo N. Neethirajan S. (2014). Biosensors for the detection of antibiotics in poultry industry—a review. *Biosensors* 4 472–493. 10.3390/bios4040472 25587435 PMC4287714

[B111] Muraoka S. Okumura R. Ogawa N. Nonaka T. Miyashita K. Senda T. (2003). Crystal structure of a full-length LysR-type transcriptional regulator, CbnR: unusual combination of two subunit forms and molecular bases for causing and changing DNA bend. *J. Mol. Biol.* 328 555–566. 10.1016/S0022-2836(03)00312-7 12706716

[B112] Newberry K. J. Brennan R. G. (2004). The structural mechanism for transcription activation by MerR family member multidrug transporter activation, N terminus. *J. Biol. Chem.* 279 20356–20362. 10.1074/jbc.M400960200 14985361

[B113] Ninfa A. J. (2010). Use of two-component signal transduction systems in the construction of synthetic genetic networks. *Curr. Opin. Microbiol.* 13 240–245. 10.1016/j.mib.2010.01.003 20149718 PMC3547608

[B114] North A. K. Klose K. E. Stedman K. M. Kustu S. (1993). Prokaryotic enhancer-binding proteins reflect eukaryote-like modularity: the puzzle of nitrogen regulatory protein C. *J. Bacteriol.* 175 4267–4273. 8331061 10.1128/jb.175.14.4267-4273.1993 PMC204865

[B115] Orth P. Schnappinger D. Hillen W. Saenger W. Hinrichs W. (2000). Structural basis of gene regulation by the tetracycline inducible Tet repressor-operator system. *Nat. Struct. Biol.* 7 215–219. 10.1038/73324 10700280

[B116] Palm G. J. Lederer T. Orth P. Saenger W. Takahashi M. Hillen W. (2007). Specific binding of divalent metal ions to tetracycline and to the tet repressor/tetracycline complex. *J. Biol. Inorg. Chem.* 13 1097–1110. 10.1007/S00775-008-0395-2 18548290

[B117] Pareja E. Pareja-Tobes P. Manrique M. Pareja-Tobes E. Bonal J. Tobes R. (2006). ExtraTrain: a database of extragenic regions and transcriptional information in prokaryotic organisms. *BMC Microbiol.* 6:29 10.1186/1471-2180-6-29 PMC1453763 16539733

[B118] Park M. Tsai S.-L. Chen W. (2013). Microbial biosensors: engineered microorganisms as the sensing machinery. *Sensors* 13 5777–5795. 10.3390/s130505777 23648649 PMC3690029

[B119] Pennella M. A. Giedroc D. P. (2005). Structural determinants of metal selectivity in prokaryotic metal-responsive transcriptional regulators. *Biometals* 18 413–428. 10.1007/s10534-005-3716-8 16158234

[B120] Pérez-Martín J. De Lorenzo V. (1995). The amino-terminal domain of the prokaryotic enhancer-binding protein XylR is a specific intramolecular repressor. *Proc. Natl. Acad. Sci. U.S.A.* 92 9392–9396. 10.1073/pnas.92.20.9392 7568139 PMC40991

[B121] Pérez-Martín J. Rojo F. de Lorenzo V. (1994). Promoters responsive to DNA bending: a common theme in prokaryotic gene expression. *Microbiol. Rev.* 58 268–290. 8078436 10.1128/mr.58.2.268-290.1994 PMC372964

[B122] Pérez-Pantoja D. Kim J. Silva-Rocha R. de Lorenzo V. (2014). The differential response of the Pben promoter of *Pseudomonas putida* mt-2 to BenR and XylS prevents metabolic conflicts in m-xylene biodegradation. *Environ. Microbiol.* 17 64–75. 10.1111/1462-2920.12443 24588992

[B123] Pohl E. Holmes R. K. Hol W. G. (1999). Crystal structure of the iron-dependent regulator (IdeR) from *Mycobacterium tuberculosis* shows both metal binding sites fully occupied. *J. Mol. Biol.* 285 1145–1156. 10.1006/jmbi.1998.2339 9887269

[B124] Priyadarshi H. Alam A. Gireesh-Babu P. Das R. Kishore P. Kumar S. (2012). A GFP-based bacterial biosensor with chromosomally integrated sensing cassette for quantitative detection of Hg(II) in environment. *J. Environ. Sci. China* 24 963–968. 10.1016/S1001-0742(11)60820-6 22893977

[B125] Qiu X. Verlinde C. L. Zhang S. Schmitt M. P. Holmes R. K. Hol W. G. (1995). Three-dimensional structure of the diphtheria toxin repressor in complex with divalent cation co-repressors. *Structure* 3 87–100. 10.1016/S0969-2126(01)00137-X 7743135

[B126] Ramos J. L. Krell T. Daniels C. Segura A. Duque E. (2009). Responses of *Pseudomonas* to small toxic molecules by a mosaic of domains. *Curr. Opin. Microbiol.* 12 215–220. 10.1016/j.mib.2009.02.001 19269884

[B127] Ramos J. L. Martínez-Bueno M. Molina-Henares A. J. Terán W. Watanabe K. Zhang X. (2005). The TetR family of transcriptional repressors. *Microbiol. Mol. Biol. Rev.* 69 326–356. 10.1128/MMBR.69.2.326-356.2005 15944459 PMC1197418

[B128] Ramos J. L. Mermod N. Timmis K. N. (1987). Regulatory circuits controlling transcription of TOL plasmid operon encoding meta-cleavage pathway for degradation of alkylbenzoates by *Pseudomonas*. *Mol. Microbiol.* 1 293–300. 10.1111/j.1365-2958.1987.tb01935.x 3448461

[B129] Ramos J. L. Stolz A. Reineke W. Timmis K. N. (1986). Altered effector specificities in regulators of gene expression: TOL plasmid xylS mutants and their use to engineer expansion of the range of aromatics degraded by bacteria. *Proc. Natl. Acad. Sci. U.S.A.* 83 8467–8471. 10.1073/pnas.83.22.8467 3022293 PMC386951

[B130] Rappas M. Schumacher J. Niwa H. Buck M. Zhang X. (2006). Structural basis of the nucleotide driven conformational changes in the AAA+ domain of transcription activator PspF. *J. Mol. Biol.* 357 481–492. 10.1016/j.jmb.2005.12.052 16430918

[B131] Rodgers M. E. Schleif R. (2009). Solution structure of the DNA binding domain of AraC protein. *Proteins* 77 202–208. 10.1002/prot.22431 19422057 PMC2745637

[B132] Ross W. Park S. J. Summers A. O. (1989). Genetic analysis of transcriptional activation and repression in the Tn21 mer operon. *J. Bacteriol.* 171 4009–4018. 2661542 10.1128/jb.171.7.4009-4018.1989 PMC210155

[B133] Ruangprasert A. Craven S. H. Neidle E. L. Momany C. (2010). Full-length structures of BenM and two variants reveal different oligomerization schemes for LysR-type transcriptional regulators. *J. Mol. Biol.* 404 568–586. 10.1016/j.jmb.2010.09.053 20932977

[B134] Sawai H. Yamanaka M. Sugimoto H. Shiro Y. Aono S. (2012). Structural basis for the transcriptional regulation of heme homeostasis in *Lactococcus lactis*. *J. Biol. Chem.* 287 30755–30768. 10.1074/jbc.M112.370916 22798069 PMC3436319

[B135] Schell M. A. (1993). Molecular biology of the LysR family of transcriptional regulators. *Annu. Rev. Microbiol.* 47 597–626. 10.1146/annurev.mi.47.100193.003121 8257110

[B136] Schiering N. Tao X. Zeng H. Murphy J. R. Petsko G. A. Ringe D. (1995). Structures of the apo- and the metal ion-activated forms of the diphtheria tox repressor from Corynebacterium diphtheriae. *Proc. Natl. Acad. Sci. U.S.A.* 92 9843–9850. 7568230 10.1073/pnas.92.21.9843 PMC40899

[B137] Schreiter E. R. Drennan C. L. (2007). Ribbon–helix–helix transcription factors: variations on a theme. *Nat. Rev. Microbiol.* 5 710–720. 10.1038/nrmicro1717 17676053

[B138] Schreiter E. R. Sintchak M. D. Guo Y. Chivers P. T. Sauer R. T. Drennan C. L. (2003). Crystal structure of the nickel-responsive transcription factor NikR. *Nat. Struct. Biol.* 10 794–799. 10.1038/nsb985 12970756

[B139] Schreiter E. R. Wang S. C. Zamble D. B. Drennan C. L. (2006). NikR-operator complex structure and the mechanism of repressor activation by metal ions. *Proc. Natl. Acad. Sci. U.S.A.* 103 13676–13681. 10.1073/pnas.0606247103 16945905 PMC1564233

[B140] Schultz S. C. Shields G. C. Steitz T. A. (1991). Crystal structure of a CAP-DNA complex: the DNA is bent by 90 degrees. *Science* 253 1001–1007 10.1126/science.1653449 1653449

[B141] Schumacher M. A. Miller M. C. Grkovic S. Brown M. H. Skurray R. A. Brennan R. G. (2002). Structural basis for cooperative DNA binding by two dimers of the multidrug-binding protein QacR. *EMBO J.* 21 1210–1218. 10.1093/emboj/21.5.1210 11867549 PMC125875

[B142] Sheikh M. A. Taylor G. L. (2009). Crystal structure of the *Vibrio cholerae* ferric uptake regulator (Fur) reveals insights into metal co-ordination. *Mol. Microbiol.* 72 1208–1220. 10.1111/j.1365-2958.2009.06718.x 19400801

[B143] Shimomura O. (1979). Structure of the chromophore of *Aequorea* green fluorescent protein. *FEBS Lett.* 104 220–222. 10.1016/0014-5793(79)80818-2

[B144] Shingler V. Franklin F. C. Tsuda M. Holroyd D. Bagdasarian M. (1989). Molecular analysis of a plasmid-encoded phenol hydroxylase from *Pseudomonas* CF600. *J. Gen. Microbiol.* 135 1083–1092. 10.1099/00221287-135-5-1083 2559941

[B145] Shi W. Dong J. Scott R. A. Ksenzenko M. Y. Rosen B. P. (1996). The role of arsenic-thiol interactions in metalloregulation of the ars operon. *J. Biol. Chem.* 271 9291–9297. 10.1074/jbc.271.16.9291 8621591

[B146] Shin H. J. (2010). Development of highly-sensitive microbial biosensors by mutation of the nahR regulatory gene. *J. Biotechnol.* 150 246–250. 10.1016/j.jbiotec.2010.09.936 20851155

[B147] Shin J.-H. Jung H. J. An Y. J. Cho Y.-B. Cha S.-S. Roe J.-H. (2011). Graded expression of zinc-responsive genes through two regulatory zinc-binding sites in Zur. *Proc. Natl. Acad. Sci. U.S.A.* 108 5045–5050. 10.1073/pnas.1017744108 21383173 PMC3064357

[B148] Siegfried K. Endes C. Bhuiyan A. F. M. K. Kuppardt A. Mattusch J. van der Meer J. R. (2012). Field testing of arsenic in groundwater samples of Bangladesh using a test kit based on lyophilized bioreporter bacteria. *Environ. Sci. Technol.* 46 3281–3287. 10.1021/es203511k 22339623

[B149] Skärfstad E. O’Neill E. Garmendia J. Shingler V. (2000). Identification of an effector specificity subregion within the aromatic-responsive regulators DmpR and XylR by DNA shuﬄing. *J. Bacteriol.* 182 3008–3016. 10.1128/JB.182.11.3008-3016.2000 10809676 PMC94483

[B150] Soisson S. M. MacDougall-Shackleton B. Schleif R. Wolberger C. (1997). The 1.6 A crystal structure of the AraC sugar-binding and dimerization domain complexed with D-fucose. *J. Mol. Biol.* 273 226–237. 10.1006/jmbi.1997.1314 9367758

[B151] Spiro S. Dixon R. (2010). *Sensory Mechanisms in Bacteria: Molecular Aspects of Signal Recognition.* Wymondham: Horizon Scientific Press.

[B152] Stevenson B. J. Yip S. H.-C. Ollis D. L. (2013). In vitro directed evolution of enzymes expressed by *E. coli* in microtiter plates. *Methods Mol. Biol.* 978 237–249. 10.1007/978-1-62703-293-3_18 23423902

[B153] Stoll K. E. Draper W. E. Kliegman J. I. Golynskiy M. V. Brew-Appiah R. A. T. Phillips R. K. (2009). Characterization and structure of the manganese-responsive transcriptional regulator ScaR. *Biochemistry* 48 10308–10320. 10.1021/bi900980g 19795834 PMC3586275

[B154] Tahlan K. Yu Z. Xu Y. Davidson A. R. Nodwell J. R. (2008). Ligand recognition by ActR, a TetR-like regulator of actinorhodin export. *J. Mol. Biol.* 383 753–761. 10.1016/j.jmb.2008.08.081 18804114

[B155] Takano H. Kondo M. Usui N. Usui T. Ohzeki H. Yamazaki R. (2011). Involvement of CarA/LitR and CRP/FNR family transcriptional regulators in light-induced carotenoid production in *Thermus thermophilus*. *J. Bacteriol.* 193 2451–2459. 10.1128/JB.01125-10 21421762 PMC3133161

[B156] Tchounwou P. B. Wilson B. Ishaque A. (1999). Important considerations in the development of public health advisories for arsenic and arsenic-containing compounds in drinking water. *Rev. Environ. Health* 14 211–229. 10.1515/REVEH.1999.14.4.211 10746734

[B157] Thöny B. Hennecke H. (1989). The -24/-12 promoter comes of age. *FEMS Microbiol. Rev.* 5 341–357. 10.1016/0168-6445(89)90028-4 2517036

[B158] Trang P. T. K. Berg M. Viet P. H. Mui N. V. van der Meer J. R. (2005). Bacterial bioassay for rapid and accurate analysis of arsenic in highly variable groundwater samples. *Environ. Sci. Technol.* 39 7625–7630. 10.1021/es050992e 16245836

[B159] Tropel D. van der Meer J. R. (2004). Bacterial transcriptional regulators for degradation pathways of aromatic compounds. *Microbiol. Mol. Biol. Rev.* 68 474–500. 10.1128/MMBR.68.3.474-500.2004 15353566 PMC515250

[B160] Troxell B. Hassan H. M. (2013). Transcriptional regulation by ferric pptake regulator (Fur) in pathogenic bacteria. *Front. Cell. Infect. Microbiol.* 3:59 10.3389/fcimb.2013.00059 PMC3788343 24106689

[B161] Turner J. S. Glands P. D. Samson A. C. Robinson N. J. (1996). Zn2+-sensing by the cyanobacterial metallothionein repressor SmtB: different motifs mediate metal-induced protein-DNA dissociation. *Nucleic Acids Res.* 24 3714–3721. 10.1093/nar/24.19.3714 8871549 PMC146171

[B162] Ulrich L. E. Koonin E. V. Zhulin I. B. (2005). One-component systems dominate signal transduction in prokaryotes. *Trends Microbiol.* 13 52–56. 10.1016/j.tim.2004.12.006 15680762 PMC2756188

[B163] Utschig L. M. Bryson J. W. O’Halloran T. V. (1995). Mercury-199 NMR of the metal receptor site in MerR and its protein-DNA complex. *Science* 268 380–385. 10.1126/science.7716541 7716541

[B164] VanZile M. L. Chen X. Giedroc D. P. (2002). Structural characterization of distinct alpha3N and alpha5 metal sites in the cyanobacterial zinc sensor SmtB. *Biochemistry* 41 9765–9775. 10.1021/bi0201771 12146942

[B165] Volkers G. Petruschka L. Hinrichs W. (2011). Recognition of drug degradation products by target proteins: isotetracycline binding to Tet repressor. *J. Med. Chem.* 54 5108–5115. 10.1021/jm200332e 21699184

[B166] Wang B. Barahona M. Buck M. (2013). A modular cell-based biosensor using engineered genetic logic circuits to detect and integrate multiple environmental signals. *Biosens. Bioelectron.* 40 368–376. 10.1016/j.bios.2012.08.011 22981411 PMC3507625

[B167] Weiss D. S. Batut J. Klose K. E. Keener J. Kustu S. (1991). The phosphorylated form of the enhancer-binding protein NTRC has an ATPase activity that is essential for activation of transcription. *Cell* 67 155–167. 10.1016/0092-8674(91)90579-N 1833069

[B168] Werlen C. Jaspers M. C. M. van der Meer J. R. (2004). Measurement of biologically available naphthalene in gas and aqueous phases by use of a *Pseudomonas putida* Biosensor. *Appl. Environ. Microbiol.* 70 43–51. 10.1128/AEM.70.1.43-51.2004 14711624 PMC321291

[B169] West A. L. St John F. Lopes P. E. M. MacKerell A. D. Pozharski E. Michel S. L. J. (2010). Holo-Ni(II)HpNikR is an asymmetric tetramer containing two different nickel-binding sites. *J. Am. Chem. Soc.* 132 14447–14456. 10.1021/ja104118r 20863122 PMC2958704

[B170] White A. Ding X. vanderSpek J. C. Murphy J. R. Ringe D. (1998). Structure of the metal-ion-activated diphtheria toxin repressor/tox operator complex. *Nature* 394 502–506. 10.1038/28893 9697776

[B171] WHO. (2012). *State of the Science of Endocrine Disrupting Chemicals – 2012.* Available at: http://www.who.int/ceh/publications/endocrine/en/ [accessed February 12, 2015].

[B172] Wiles S. Robertson B. D. Frankel G. Kerton A. (2009). Bioluminescent monitoring of in vivo colonization and clearance dynamics by light-emitting bacteria. *Methods Mol. Biol.* 574 137–153. 10.1007/978-1-60327-321-3_12 19685306

[B173] Wilkinson S. P. Grove A. (2006). Ligand-responsive transcriptional regulation by members of the MarR family of winged helix proteins. *Curr. Issues Mol. Biol.* 8 51–62. 16450885

[B174] Willems A. R. Tahlan K. Taguchi T. Zhang K. Lee Z. Z. Ichinose K. (2008). Crystal structures of the *Streptomyces coelicolor* TetR-like protein ActR alone and in complex with actinorhodin or the actinorhodin biosynthetic precursor (S)-DNPA. *J. Mol. Biol.* 376 1377–1387. 10.1016/j.jmb.2007.12.061 18207163

[B175] Wisedchaisri G. Chou C. J. Wu M. Roach C. Rice A. E. Holmes R. K. (2007). Crystal structures, metal activation, and DNA-binding properties of two-domain IdeR from *Mycobacterium tuberculosis*. *Biochemistry* 46 436–447. 10.1021/bi0609826 17209554

[B176] Wisedchaisri G. Holmes R. K. Hol W. G. (2004). Crystal structure of an IdeR-DNA complex reveals a conformational change in activated IdeR for base-specific interactions. *J. Mol. Biol.* 342 1155–1169. 10.1016/j.jmb.2004.07.083 15351642

[B177] Xiong A. Gottman A. Park C. Baetens M. Pandza S. Matin A. (2000). The EmrR protein represses the *Escherichia coli* emrRAB multidrug resistance operon by directly binding to its promoter region. *Antimicrob. Agents Chemother.* 44 2905–2907. 10.1128/AAC.44.10.2905-2907.2000 10991887 PMC90178

[B178] Xu Y. Heath R. J. Li Z. Rock C. O. White S. W. (2001). The FadR.DNA complex. Transcriptional control of fatty acid metabolism in *Escherichia coli. J. Biol. Chem.* 276 17373–17379. 10.1074/jbc.M100195200 11279025

[B179] Xue H. Shi H. Yu Z. He S. Liu S. Hou Y. (2014). Design, construction, and characterization of a set of biosensors for aromatic compounds. *ACS Synth. Biol.* 3 1011–1014. 10.1021/sb500023f 25524112

[B180] Yamaguchi A. Adachi K. Akasaka T. Ono N. Sawai T. (1991). Metal-tetracycline/H+ antiporter of *Escherichia coli* encoded by a transposon Tn10. Histidine 257 plays an essential role in H+ translocation. *J. Biol. Chem.* 266 6045–6051. 1848846

[B181] Yang J. Tauschek M. Robins-Browne R. M. (2011). Control of bacterial virulence by AraC-like regulators that respond to chemical signals. *Trends Microbiol.* 19 128–135. 10.1016/j.tim.2010.12.001 21215638

[B182] Yang S. Gao Z. Li T. Yang M. Zhang T. Dong Y. (2013). Structural basis for interaction between *Mycobacterium smegmatis* Ms6564, a TetR family master regulator, and its target DNA. *J. Biol. Chem.* 288 23687–23695. 10.1074/jbc.M113.468694 23803605 PMC3745316

[B183] Ye J. Kandegedara A. Martin P. Rosen B. P. (2005). Crystal structure of the *Staphylococcus aureus* pI258 CadC Cd(II)/Pb(II)/Zn(II)-responsive repressor. *J. Bacteriol.* 187 4214–4221. 10.1128/JB.187.12.4214-4221.2005 15937183 PMC1151749

[B184] Yokobayashi Y. Weiss R. Arnold F. H. (2002). Directed evolution of a genetic circuit. *Proc. Natl. Acad. Sci. U.S.A.* 99 16587–16591. 10.1073/pnas.252535999 12451174 PMC139187

[B185] Zeng Q. Stålhandske C. Anderson M. C. Scott R. A. Summers A. O. (1998). The core metal-recognition domain of MerR. *Biochemistry* 37 15885–15895. 10.1021/bi9817562 9843394

[B186] Zhang F. Keasling J. (2011). Biosensors and their applications in microbial metabolic engineering. *Trends Microbiol.* 19 323–329. 10.1016/j.tim.2011.05.003 21664818

[B187] Zhao H. Volkov A. Veldore V. H. Hoch J. A. Varughese K. I. (2010). Crystal structure of the transcriptional repressor PagR of *Bacillus anthracis*. *Microbiol. Read. Engl.* 156 385–391. 10.1099/mic.0.033548-0 PMC2828352 19926656

